# Age and Gender Affect the Composition of Fungal Population of the Human Gastrointestinal Tract

**DOI:** 10.3389/fmicb.2016.01227

**Published:** 2016-08-03

**Authors:** Francesco Strati, Monica Di Paola, Irene Stefanini, Davide Albanese, Lisa Rizzetto, Paolo Lionetti, Antonio Calabrò, Olivier Jousson, Claudio Donati, Duccio Cavalieri, Carlotta De Filippo

**Affiliations:** ^1^Department of Computational Biology, Research and Innovation Centre, Fondazione Edmund MachSan Michele all' Adige, Italy; ^2^Centre for Integrative Biology, University of TrentoTrento, Italy; ^3^Department of Neuroscience, Psychology, Drug Research and Child Health, Meyer Children's Hospital, University of FlorenceFlorence, Italy; ^4^Gastroenterology Unit, Department of Experimental and Clinical Biomedical Sciences, University of FlorenceFlorence, Italy; ^5^Department of Biology, University of Florence, Sesto FiorentinoFlorence, Italy; ^6^Institute of Biometeorology, National Research CouncilFlorence, Italy

**Keywords:** commensal fungi, human gut mycobiota, antifungal resistance, fungal metagenomics, fungi-host interactions

## Abstract

The fungal component of the human gut microbiota has been neglected for long time due to the low relative abundance of fungi with respect to bacteria, and only recently few reports have explored its composition and dynamics in health or disease. The application of metagenomics methods to the full understanding of fungal communities is currently limited by the under representation of fungal DNA with respect to the bacterial one, as well as by the limited ability to discriminate passengers from colonizers. Here, we investigated the gut mycobiota of a cohort of healthy subjects in order to reduce the gap of knowledge concerning fungal intestinal communities in the healthy status further screening for phenotypical traits that could reflect fungi adaptation to the host. We studied the fecal fungal populations of 111 healthy subjects by means of cultivation on fungal selective media and by amplicon-based ITS1 metagenomics analysis on a subset of 57 individuals. We then characterized the isolated fungi for their tolerance to gastrointestinal (GI) tract-like challenges and their susceptibility to antifungals. A total of 34 different fungal species were isolated showing several phenotypic characteristics associated with intestinal environment such as tolerance to body temperature (37°C), to acidic and oxidative stress, and to bile salts exposure. We found a high frequency of azoles resistance in fungal isolates, with potential and significant clinical impact. Analyses of fungal communities revealed that the human gut mycobiota differs in function of individuals' life stage in a gender-related fashion. The combination of metagenomics and fungal cultivation allowed an in-depth understanding of the fungal intestinal community structure associated to the healthy status and the commensalism-related traits of isolated fungi. We further discussed comparatively the results of sequencing and cultivation to critically evaluate the application of metagenomics-based approaches to fungal gut populations.

## Introduction

The human gut is a complex ecological niche in which archaea, bacteria, protozoa, fungi, and viruses co-exist in close association with the host (Reyes et al., 2010; Arumugam et al., 2011; Human Microbiome Project Consortium, 2012). Even if it has been estimated that the number of bacteria hugely outreaches the number of fungi in the gastrointestinal (GI) tract (Huffnagle and Noverr, 2013), fungi play a relevant role in the physiology of the human host (Oever and Netea, 2014; Underhill and Iliev, 2014). Recent studies showed that, while the composition of the bacterial community is relatively stable over time, the fungal population inhabiting the murine gut undergoes significant changes during the animal's lifetime (Dollive et al., 2013). This brought to the conclusion that gut fungal populations are more variable than bacterial ones and that their composition may be influenced by environmental fungi (Underhill and Iliev, 2014). Despite evidence that fungi inhabit the mammalian GI tract and interact with the host immune system (Romani, 2011; Rizzetto et al., 2014; Underhill and Iliev, 2014), the composition and characteristics of the mycobiota in healthy hosts have been poorly explored. The prevalent interest in describing pathogenic fungi, their phenotypes and the process by which they establish the infection is one of the major cause that brought to neglect the harmless part of the commensal fungal population. Despite this topic has been only marginally explored to date, it has been shown that mucosal fungi are able to modulate both the innate and adaptive immune responses (Romani, 2011; Rizzetto et al., 2014; Underhill and Iliev, 2014) thus supporting the need to further study the whole gut mycobiota. Furthermore, alterations of the gut mycobiota have been associated to different pathologies ranging from metabolic disorders (obesity) to colorectal adenomas and Inflammatory Bowel Diseases (IBDs) (Luan et al., 2015; Mar Rodriguez et al., 2015; Sokol et al., 2016). A recent study showed the association of IBDs to alteration of the gut mycobiota. In particular Sokol and colleagues showed that IBD patients bear a smaller proportion of *Saccharomyces cerevisiae* and higher of *Candida albicans* compared to healthy subjects. In addition, they highlighted the existence in Crohn's disease of interconnected alterations between bacterial and fungal communities (Sokol et al., 2016). However, the role of the gut mycobiota in the maintenance of health it is still far from being well-understood because the studies carried out so far focused on disease-causing taxa. Nevertheless, some yeasts have been clinically prescribed for a long time because of their potential probiotic properties, suggesting a beneficial role of some fungi for host health. A great example of “beneficial” fungus is represented by *S. cerevisiae* var. *boulardii*, used for the relief of gastroenteritis (Hatoum et al., 2012). In order to reduce the gap of knowledge concerning the gut mycobiota and its interplay with the host, we characterized the gut mycobiota composition of a cohort of healthy subjects by means of metagenomics, fungal cultivation, and phenotypic assays.

## Materials and methods

### Study participants

Fecal samples were collected from 111 Italian healthy volunteers (49 male and 62 female, average age, 10 ± 8.2; Table 1) and analyzed within 24 h. Written informed consent has been obtained from all the enrolled subjects or tutors in accordance with the guidelines and regulations approved by the Research Ethical Committees of the Meyer Children's Hospital and the Azienda Ospedaliera Careggi, Florence. All the subjects enrolled were non-smokers, followed a Mediterranean-based diet and they did not take antibiotics, antifungals or probiotics in the 6 months prior to sample collection. None of the participants had any history of GI abnormalities.

**Table 1:** **Characteristics of the study participants**.

**Age group (year)**		**Infants (0–2)**	**Children (3–10)**		**Adolescents (11–17)**		**Adults (≥18)**	**All subjects**
**Number of subjects**		18	48		24		21	111
**% with fungi**		88.9	83.3		70.8		76.2	80.2
**Subject ID**	**Gender**	**Age (year)**	**Subject ID**	**Gender**	**Age (year)**	**Subject ID**	**Gender**	**Age (year)**
HS1	M	5	HS38^*^	F	25	HS75	F	1
HS2	M	5	HS39^*^	F	27	HS76^*^	M	1
HS3	M	14	HS40^*^	M	27	HS77	F	4
HS4	M	1	HS41^*^	F	24	HS78^*^	M	12
HS5	F	20	HS42^*^	F	24	HS79^*^	M	0.1
HS6	F	20	HS43^*^	M	26	HS80	F	0.1
HS7	F	20	HS44^*^	F	24	HS81	F	7
HS8	M	5	HS45^*^	F	6	HS82^*^	M	10
HS9	M	14	HS46^*^	F	6	HS83^*^	M	12
HS10^*^	F	2	HS47^*^	F	10	HS84	F	6
HS11	M	16	HS48^*^	F	2.5	HS85	F	10
HS12	M	15	HS49^*^	M	2.5	HS86^*^	M	7
HS13^*^	F	18	HS50^*^	F	1.5	HS87^*^	M	9
HS14	F	0.3	HS51^*^	F	8	HS88^*^	M	7
HS15^*^	F	11	HS52^*^	F	23	HS89^*^	M	12
HS16	M	14	HS53^*^	F	23	HS90	F	8
HS17	M	15	HS54	M	2	HS91	F	2
HS18	M	11	HS55^*^	M	2	HS92	F	12
HS19	F	3	HS56^*^	M	2	HS93	F	4
HS20^*^	F	4	HS57	F	12	HS94	F	4
HS21^*^	F	5	HS58	F	3	HS95	F	10
HS22^*^	F	15	HS59^*^	M	5	HS96	F	12
HS23^*^	F	11	HS60	F	3	HS97^*^	M	6
HS24	M	15	HS61^*^	M	2	HS98	F	16
HS25	M	7	HS62	F	4	HS99	F	3
HS26	M	3	HS63^*^	M	5	HS100^*^	M	0.1
HS27^*^	F	9	HS64	F	3	HS101^*^	M	4
HS28	M	5	HS65^*^	M	5	HS102	F	13
HS29^*^	F	16	HS66^*^	M	0.1	HS103^*^	M	7
HS30^*^	F	12	HS67	F	1	HS104^*^	M	4
HS31^*^	F	24	HS68	F	4	HS105	F	8
HS32^*^	F	32	HS69^*^	M	6	HS106	F	5
HS33^*^	F	32	HS70	F	11	HS107	M	13
HS34^*^	F	25	HS71^*^	M	1	HS108	M	4.5
HS35^*^	F	26	HS72	F	10	HS109	M	1
HS36^*^	M	20	HS73	F	4	HS110	M	12
HS37^*^	F	28	HS74^*^	M	6	HS111	M	18

* *Samples analyzed also by mean of amplicon-based ITS1metagenomics*.

### Isolation and identification of cultivable fungal species from Feces

Stool samples were diluted in sterile Ringer's solution and plated on solid YPD medium (1% Yeast extract, 2% Bacto-peptone, 2% D-glucose, 2% agar) supplemented with 25 U/ml of penicillin, 25 μg/ml of streptomycin (Sigma-Aldrich) and incubated aerobically at 27°C for 3–5 days. All fungal isolates grown on the selective medium were further isolated to obtain single-cell pure colonies. Genomic DNA was extracted from pure cultures of isolated colonies as previously described (Hoffman and Winston, 1987). Strains were identified by amplification and sequencing of the ribosomal Internal Transcribed Spacer (ITS) region, using ITS1 (5′-GTTTCCGTAGGTGAACTTGC-3′) and ITS4 (5′-TCCTCCGCTTATTGATATGC-3′) primers, as previously described (Sebastiani et al., 2002). Fungal isolates were identified by using the BLAST algorithm in the NCBI database (minimum 97% sequence similarity and 95% coverage with a described species).

### Phenotypical characterization of fungal isolates

Fungal isolates were tested for phenotypical features that could be related to the ability of colonization and persistence in the human gut. Cell growth in liquid media was monitored by optical density measurement at 630 nm with a microplate reader (Synergy2, BioTek, USA) after 48 h of incubation under tested conditions. Three independent replicates were performed for each test.

#### Growth at supra optimal temperatures

Fungal isolates (~10^5^ cells/ml) were grown at supra optimal temperatures in liquid YPD medium (40, 42, 44, and 46°C).

#### pH impact on growth

Fungal isolates (~10^5^ cells/ml) were grown at 37°C in liquid YPD medium at pH 2.0 and pH 3.0 adding hydrochloric acid/potassium chloride and citrate buffers, respectively, to test their ability to resist to the acidic environments encountered during GI tract passage.

#### Tolerance to bile acids

Fungal isolates (~10^5^ cells/ml) were grown in liquid YPD medium at 37°C in the presence of three different concentrations of bile [Ox-bile, Sigma-Aldrich; 0.5, 1, and 2% (w/v)] mimicking the physiological intestinal settings (Noriega et al., 2004).

#### Resistance to oxidative stress

Fungal resistance to oxidative stress was evaluated by measuring the inhibition halo induced by the treatment of fungal strains (~10^7^ cells/ml) grown on YPD solid medium with 0.5 mM hydrogen peroxide (H_2_O_2_). The percentage of sensitivity to oxidative stress was calculated as the deviation of the inhibition halo diameter (Ø) from that of the environmental, oxidative stress sensitive M28-4D *S. cerevisiae* strain (Cavalieri et al., 2000) according to the following formula: [(Ø sample—Ø M28-4D)/Ø M284D]^*^100.

#### Invasive growth

The ability of fungal strains to penetrate the YPD solid medium was tested as previously described (Vopalenska et al., 2005). M28-4D and BY4742 *S. cerevisiae* strains, known to be invasive and non-invasive, respectively, have been used as controls. The strain invasiveness was assigned with scores from 3 (highly invasive) to 0 (non-invasive).

#### Hyphal formation

Fungal cells (~10^5^ cells/ml) were grown for 7 days in liquid YPD and YNB media [0.67% Yeast Nitrogen Base w/o aminoacids and (NH_4_)_2_SO_4_ (Sigma-Aldrich), 2% glucose], both at 27 and 37°C in order to evaluate hyphae or pseudohyphae formation. Formation of hyphae was inspected by optical microscope observation with a Leica DM1000 led instrument (magnification 40x and 100x).

### Antifungal susceptibility testing

All fungal isolates were tested for susceptibility to fluconazole, itraconazole, and 5-flucytosine (Sigma-Aldrich) by Minimum Inibitory Concentration (MIC) assays according to the European Committee on Antimicrobial Susceptibility Testing (EUCAST) recommendations (Rodriguez-Tudela et al., 2008a,b). Clinical and Laboratory Standards Institute (CLSI) clinical breakpoints (CBPs) were used to evaluate the antifungal resistance (Pfaller and Diekema, 2012; Castanheira et al., 2014). CBPs have not been established for non-*Candida* yeasts and non-*Aspergillus* molds, however have been used as a proxy for the evaluation of antifungals susceptibility in such isolates.

### DNA extraction and PCR amplification of fungal ITS1 rDNA region

DNA extraction from fecal samples (250 mg) was performed using the FastDNA™ SPIN Kit for Feces (MP-Biomedicals, USA) following manufacturer's instructions. DNA quality was checked on 1% agarose gel TAE 1X and quantified with a NanoDrop® spectrophotometer. For each sample, fungal ITS1 rDNA region was amplified using a specific fusion primer set coupled with forward primer 18SF (5′-GTAAAAGTCGTAACAAGGTTTC-3′) and reverse primer 5.8S1R (5′-GTTCAAAGAYTCGATGATTCAC-3′; Findley et al., 2013) containing adaptors, key sequence and barcode (Multiple IDentifier) sequences as described by the 454 Sequencing System Guidelines for Amplicon Experimental Design (Roche, Switzerland). The PCR reaction mix contained 1X FastStart High Fidelity PCR buffer, 2 mM MgCl_2_, 200 μM of dNTPs, 0.4 μM of each primer (PRIMM, Italy), 2.5 U of FastStart High Fidelity Polymerase Blend, and 100 ng of gDNA as template. Thermal cycling conditions used were 5 min at 95°C, 35 cycles of 45 s at 95°C, 45 s at 56°C, and 1.30 min at 72°C followed by a final extension of 10 min at 72°C. All PCR experiments were carried out in triplicates using a Veriti® Thermal Cycler (Applied Biosystems, USA).

### Library construction and pyrosequencing

The PCR products obtained were analyzed by gel electrophoresis and cleaned using the AMPure XP beads kit (Beckman Coulter, USA) following the manufacturer's instructions, quantified via quantitative PCR using the Library quantification kit—Roche 454 titanium (KAPA Biosystems, USA) and pooled in equimolar way in a final amplicon library. The 454 pyrosequencing was carried out on the GS FLX+ system using the XL+ chemistry following the manufacturer's recommendations (Roche, Switzerland).

### Data analysis

Pyrosequencing resulted in a total of 1.337.184 reads with a mean of 19.379 ± 13.334 sequences *per* sample. Raw 454 files were demultiplexed using the Roche's sff file software and submitted to the European Nucleotide Archive with accession number PRJEB11827 (http://www.ebi.ac.uk/ena/data/view/PRJEB11827). Sample accessions and metadata are available in Supplementary Table S1. Reads were pre-processed using the MICCA pipeline (Albanese et al., 2015) (http://www.micca.org). Forward and reverse primers trimming and quality filtering were performed using micca-preproc. *De-novo* sequence clustering, chimera filtering, and taxonomy assignment were performed by micca-otu-*denovo*: Operational Taxonomic Units (OTUs) were assigned by clustering the sequences with a threshold of 97% pairwise identity and their representative sequences were classified using the RDP classifier version 2.8 (Wang et al., 2007) against the UNITE fungal ITS database (Koljalg et al., 2013). *De novo* multiple sequence alignment was performed using T-Coffee (Notredame et al., 2000). Fungal taxonomy assignments were then manually curated using BLASTn against the GenBank's database for accuracy. High quality fungal sequences were detected in all samples. Furthermore, the sequences belonging to Agaricomycetes [unlikely to be residents of the human gut due to their ecology Hibbett, 2006] were manually filtered out.

The phylogenetic tree was inferred by using micca-phylogeny (Price et al., 2010). Rarefaction analysis resulted in a sequencing depth adequate to capture the ecological diversity of the samples up to saturation. Sampling heterogeneity was reduced by rarefaction. *Alpha* and *beta*-diversity estimates were computed using the phyloseq R package (McMurdie and Holmes, 2013). PERMANOVA (Permutational multivariate analysis of variance) was performed using the adonis() function of the *vegan* R package with 999 permutations. Permutations have been constrained within age groups (corresponding to 0–2, 3–10, 11–17, and >18 y/o) or gender to reduce possible biases related to the unequal age and gender distributions among subjects using the “strata” argument within the adonis() function. Two-sided, unpaired Welch t-statistics were computed using the function mt() in the phyloseq library and the *p*-values were adjusted for multiple comparison controlling the family-wise Type I error rate (minP procedure; Westfall and Young, 1993). Wilcoxon rank-sum tests and Spearman's correlations were performed using the R software (Team, 2014) through the *stats* R package (version 3.1.2) and the *psych* R package (Revelle, 2013), respectively. *p*-values have been corrected for multiple comparison by using the false discovery rate correction (Benjamini and Hochberg, 1995).

## Results

### Cultivable gut mycobiota

The cultivable gut mycobiota of 111 healthy volunteers was investigated through isolation in selective media. Fungi were detected in more than 80% of subjects leading to the identification of 349 different isolates (Supplementary Table S2). Thirty-four different fungal species were detected at different frequencies of isolation (Table 2) among which *Aspergillus glaucus, Candida albicans, Candida deformans, Candida fermentati, Candida glabrata, Candida intermedia, Candida lusitaniae, Candida metapsilosis, Candida parapsilosis, Candida pararugosa, Candida tropicalis, Candida zelanoydes, Cryptococcus saitoi, Lichtheimia ramosa, Mucor circinelloides, Pleurostomophora richardsiae, Rhodotorula mucilaginosa, Trichosporon asahii, Yarrowia lipolytica*. These species were previously found in different human body sites, including the GI tract as commensal or opportunistic pathogens (Araujo et al., 2007; Johnson, 2009; Alastruey-Izquierdo et al., 2010; Kurtzman et al., 2011; Levenstadt et al., 2012; Gouba et al., 2014; Lee et al., 2014; Rizzetto et al., 2014). We also isolated the environmental fungi *Aspergillus pseudoglaucus, Eurotium amstelodami, Eurotium rubrum, Penicillium brevicompactum, Penicillium paneum, Penicillium crustosum, Pichia caribbica, Pichia fermentans, Pichia kluyveri, Pichia manshurica, Rhodosporidium kratochvilovae, Saccharomyces cerevisiae, Starmerella bacillaris*, and *Torulaspora delbrueckii*. Such species were previously found in fermentations, oenological samples (Chitarra et al., 2004; Butinar et al., 2005; Kurtzman et al., 2011; Barata et al., 2012; Bezerra-Bussoli et al., 2013; Tristezza et al., 2013; Vardjan et al., 2013; Belda et al., 2015; de Melo Pereira et al., 2014; Santini et al., 2014; Wang et al., 2014) and rarely found in clinical samples (de la Camara et al., 1996; Kaygusuz et al., 2003; Butinar et al., 2005; Rizzetto et al., 2014). The 39.8% of subjects showed at least one *C. albicans* isolate, which resulted in the most common yeast species found in our samples, in line with previous reports on the gut mycobiota of healthy subjects (Khatib et al., 2001; Bougnoux et al., 2006).

**Table 2:** **Fungal isolates and frequencies of isolation**.

**Species**	**%**	**Species**	**%**
*Candida albicans*	39.8	*Rhodosporidium kratochvilovae*	0.57
*Rhodotorula mucilaginosa*	12.6	*Trichosporon asahii*	0.57
*Candida parapsilosis*	12.3	*Yarrowia lipolytica*	0.57
*Torulaspora delbrueckii*	6.59	*Aspergillus cristatus*	0.28
*Pichia fermentans*	4.29	*Candida deformans*	0.28
*Penicillium brevicompactum*	3.72	*Candida fermentati*	0.28
*Pichia manshurica*	3.43	*Candida glabrata*	0.28
*Pichia kluyveri*	2.86	*Candida intermedia*	0.28
*Candida lusitaniae*	2.58	*Candida metapsilosis*	0.28
*Pennicillium crustosum*	1.43	*Candida tropicalis*	0.28
*Saccharomyces cerevisiae*	1.14	*Candida zelanoydes*	0.28
*Penicillium paneum*	0.58	*Eurotium amstelodami*	0.28
*Aspergillus glaucus*	0.57	*Eurotium rubrum*	0.28
*Aspergillus pseudoglaucus*	0.57	*Lichtheimia ramose*	0.28
*Candida pararugosa*	0.57	*Pichia carribica*	0.28
*Cryptococcus saitoi*	0.57	*Pleurostomophora richardsiae*	0.28
*Mucor circinelloides*	0.57	*Starmerella bacillaris*	0.28

**Figure 1:**
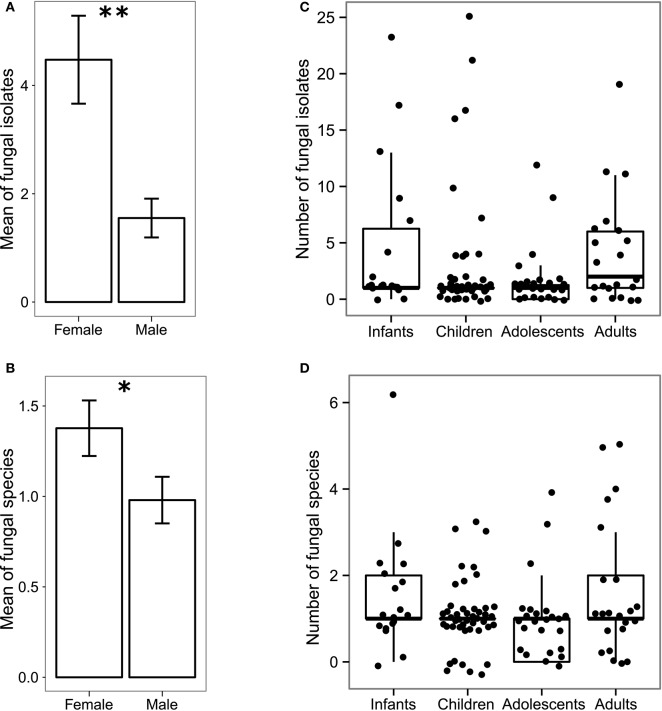
**Gender-related and age-related differences in the gut mycobiota of 111 healthy volunteers**. Histogram of the mean of **(A)** abundances and **(B)** the richness ± standard error of fungal isolates in female and male subjects; box-plot representation of the **(C)** abundance and **(D)** richness of fungal isolates in different age groups i.e., infants (0–2 years old), children (3–10 years old), adolescents (11–17 years old) and adults (≥18 years old). ^**^p < 0.005, ^*^p < 0.05, Wilcoxon rank-sum test.

Population level analysis of the cultivable gut mycobiota revealed significant gender-related differences, with female subjects showing a higher number of fungal isolates (*p* < 0.005, Wilcoxon rank-sum test; Figure 1A) and fungal species (*p* < 0.05, Wilcoxon rank-sum test; Figure 1B) compared to male subjects (not related to individual's age) while we did not observed significant differences in the fungal population among the investigated age groups (Figures 1C,D). Finally, no species *per se* was responsible for these differences, as indicated by the fact that we did not find significant differences between individual species abundances in male and female subjects for any investigated age group.

### Fungal gut metagenomics

**Figure 2:**
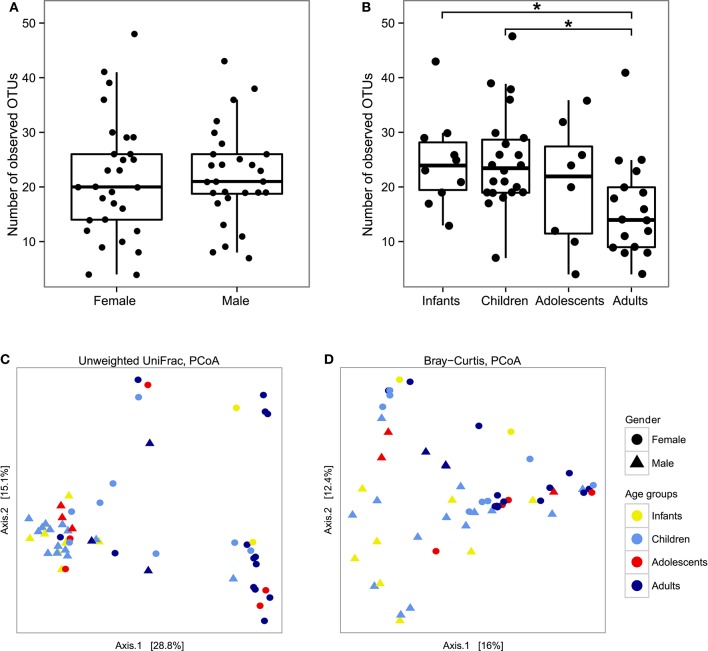
**Box-plot representation of fungal ***alpha***-diversity measures using the number of observed OTUs between (A) genders or (B) age groups and measures of fungal ***beta***-diversity by PCoA of the between samples distances measured using (C) the unweighted UniFrac distance and (D) the Bray-Curtis dissimilarity**. ^*^p < 0.05, Wilcoxon rank-sum test.

**Figure 3:**
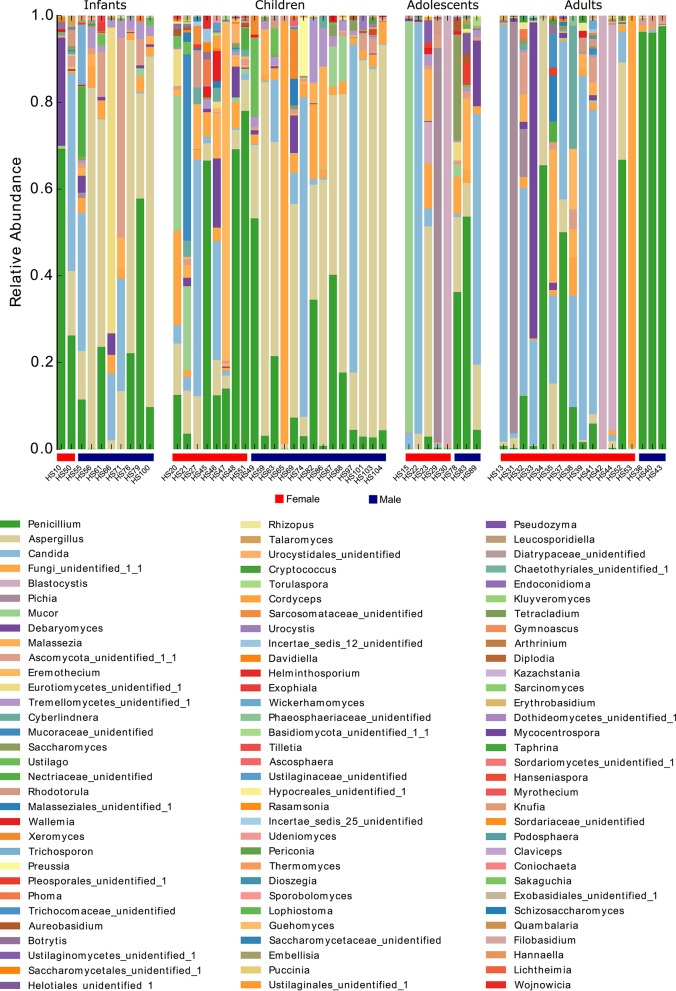
**Stacked bar-plot representation of the relative abundances at the genus level of the fecal mycobiota of healthy subjects from metagenomics analysis distributed according to individuals' life stage and gender**.

To better characterize the intestinal fungal community structure associated to our cohort of healthy subjects we further analyzed a subset of these subjects (57 subjects, 29 females, and 28 males, average age 12 ± 9.5) by means of amplicon-based ITS1 targeted metagenomics, looking at gender and age groups differences. The analysis led to the identification of 68 fully classified (to the genus level) fungal taxa and 26 taxa only partially classified (of which 2 classified to the phylum level, 5 classified to the order level, 9 classified to the class level, and 9 classified to the family level). Measurements of the fungal richness within each sample i.e., the *alpha*-diversity (see Materials and Methods), revealed no significant differences among male and female subjects (Figure 2A), differently from the above finding based on the culture-based analysis in which we observed an increased number of intestinal fungal species in females compared to males (Figure 1B). Furthermore, we observed that infants and children harbor a higher fungal richness compared to adults as indicated by the number of the observed OTUs (*p* < 0.05, Wilcoxon rank-sum test, Figure 2B). The analysis of *beta*-diversity identified significant differences in the composition of the gut mycobiota among gender and age groups. PCoA (Principal Coordinates Analysis) revealed that samples cluster by gender, based on the unweighted UniFrac distance and the Bray-Curtis dissimilarity (*p* < 0.05, PERMANOVA; Figures 2C,D, Supplementary Table S3) and by age groups, based on the unweighted UniFrac distance (*p* < 0.05, PERMANOVA; Figure 2C, Supplementary Table S3). We calculated PERMANOVAs constraining permutations within levels (gender or age groups) to avoid biases related to the unequal distribution of genders among age groups and *vice-versa*. Genus level analysis showed *Penicillium, Aspergillus*, and *Candida* as the most abundant genera in this subset of subjects (22.3, 22.2, and 16.9%, respectively; Figure 3, Supplementary Table S4). We further observed that *Aspergillus* and Tremellomycetes_unidentified_1 were significantly more abundant in male than female subjects (*p* < 0.05, Welch *t*-test) and in children than adults (*p* < 0.05, Welch *t*-test). To note, the latter result could be biased by the unbalanced distribution of male and female subjects in children and adults groups (14/22 male children and 3/17 male adults). Furthermore, the genus *Penicillium* was significantly more abundant in infants than adults (*p* < 0.05, Welch *t*-test). Interestingly, we identified sequences belonging to the single-cell protozoa *Blastocystis*, eukaryotes abundant in the human gut microbiota (Scanlan and Marchesi, 2008), only in adolescent and adult females (Figure 3, Supplementary Table S4) that could potentially be due to exposure to animals (Scanlan et al., 2014).

### Phenotyping the gut mycobiota

The characterization of phenotypic features of the isolates related to the ability to survive and colonize the human gut was performed to estimate if such isolates were commensals adapted to this ecological niche or passengers introduced through the diet and delivered with the feces.

We therefore investigated the isolates' resistance by a series of assays mimicking the conditions that fungal isolates face during passage through the human GI tract. In addition to the fact that the human body temperature (37°C) is higher than the optimum for most fungal species, in the GI tract fungi are also exposed to acidic and oxidative environments and to bile salts, produced by the liver and secreted into the duodenum, exposing the microorganisms to oxidative stress and DNA damage (Kandell and Bernstein, 1991).

**Figure 4:**
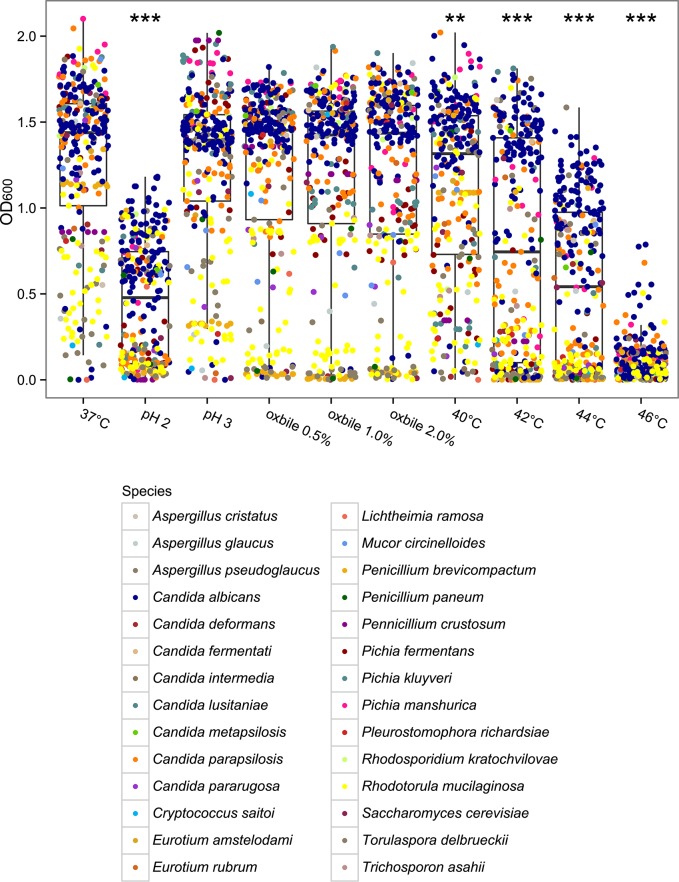
**Box-plot representation of the comparison of fungal isolates growth ability at 37°C (control condition) vs. different stressful conditions mimicking the gastrointestinal tract challenges**. ^**^p < 0.0005, ^***^p < 0.0001, Wilcoxon rank-sum test.

The majority of the isolates were found to tolerate acidic conditions (58.9 and 94.8% of isolates were able to grow at pH 2 and pH 3, respectively) and oxidative stress (85.7% of the isolates showed higher tolerance compared to environmental M28 *S. cerevisiae* strain), both conditions are characteristic of the gut environment. Tolerance to physiological concentrations of bile acids was also observed (89.8, 87.5, and 85.7% of fungal isolates were able to grow in presence of ox-bile 0.5, 1, and 2%, respectively) as well as the ability to grow at *supra* optimal temperatures with almost all the isolates (99.4%) being able to grow at 37°C (Supplementary Table S2). The comparison of the growth ability of such isolates at pH 3 and at growing concentrations of ox-bile (i.e., 0.5, 1.0, and 2.0% ox-bile) with respect to the control growth condition (37°C, no bile, pH 6.5) revealed that these stressful conditions do not significantly affect the growth ability of the fungal isolates (Figure 4). By contrast, a significant growth reduction was observed when comparing the isolated grown at pH 2 with respect to the control growth condition (*p* < 0.0001, Wilcoxon rank-sum test; Figure 4). As expected, a progressive reduction of growth ability was observed in correspondence of incubation temperature increase (i.e., from 40 to 46°C) for all the tested isolates (*p* < 0.0005, Wilcoxon rank-sum test; Figure 4).

**Figure 5:**
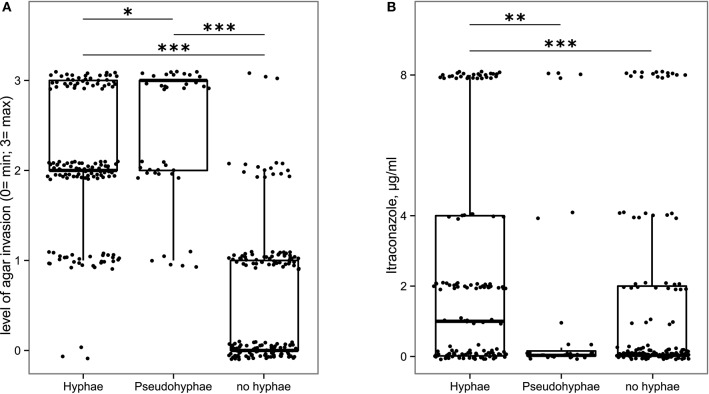
**Box-plot representation of fungal isolates able (or not) to produce hyphae or pseudohyphae in relationship with (A) their ability to be invasive on YPD solid medium, (B) their resistance to itraconazole**. ^*^p < 0.05, ^**^p < 0.005, ^***^p < 0.001, Wilcoxon rank-sum test.

In addition to the ability of fungal isolates to tolerate the intestinal environmental stresses, we also explored their ability to undergo phenotypic changes favoring their persistence within the human gut. Among these, we assessed the formation of hyphae and the ability to penetrate the solid growth medium, thus to adhere to host tissues. The 56.9% of fungal isolates was able to form hyphae or pseudohyphae (Supplementary Table S2). In addition, the morphotype switch to hyphae and pseudo-hyphae was related to the isolates' invasiveness, with hyphae and pseudohyphae-forming isolates being the most invasive (Figure 5A), suggesting that such isolates may be able to adhere to or invade the host tissues. Furthermore, we observed that hyphae-forming isolates are significantly more resistant to itraconazole than pseudohyphae-forming isolates and isolates unable to form hyphae (*p* < 0.05, Wilcoxon rank-sum test; Figure 5B). These phenotypic traits in conditions of altered immune system or in association with intestinal dysbiosis, could represent a pathogenic potential for the host.

**Figure 6:**
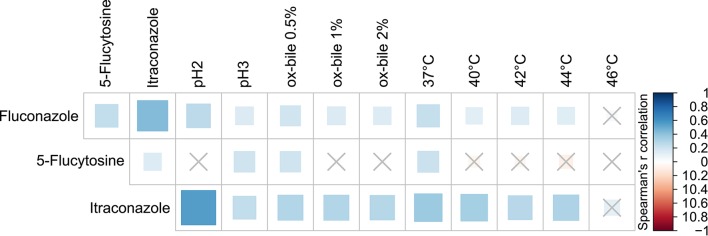
**Spearman's ***r*** correlation analysis between antifungals susceptibility and growth ability of the tested fungal isolates under different stress conditions**. Solid squares represent the degree of correlation among the variables taken into account. Crossed squares indicate non-significant correlations; significant results with *p* < 0.05.

It is now recognized that inappropriate antifungal use contributes to the increase in microbial antifungal resistance, complicating therapeutic intervention, and the eventual eradication of pathogens (Chen et al., 2010; Arendrup et al., 2011). Due to the relevance of such aspect and its impact on clinical studies, we tested all fungal isolates for their susceptibility to the widely therapeutically used azoles, fluconazole, and itraconazole (Martin, 2000) as well as the non-azole antifungal 5-flucytosine (Vermes et al., 2000). A total of 31.5% of the isolates were resistant to fluconazole and, as expected, similar levels of itraconazole resistance were found (for 39.2% of the isolates the MIC was ≥1 μg/ml; Supplementary Table S2). Previous studies have indeed suggested that cross-resistance may occur between fluconazole and other azole compounds (i.e., itraconazole) (Pfaller et al., 2006) and we further confirmed such observations with the finding of a significant positive correlation between the isolates resistance to these two antifungals (Spearman's *r* = 0.43, *p* < 0.05; Figure 6). Most of the isolates (99.34%) showed high susceptibility to 5-flucytosine with most MIC values ≤0.125 μg/ml (Supplementary Table S2). Among the 9 most abundant species (at least 6 isolates per species), *C. albicans, Pichia* spp. and *Rhodotorula mucillaginosa* showed the highest resistance to fluconazole, with MIC_90_ > 64 μg/ml (Table 3). Furthermore, it is worth to note that resistance to tested antifungals is positively correlated with the ability of strains to grow under stressful conditions, such as *supra* optimal temperature, acidic conditions, and bile salts exposure (*p* < 0.05, Spearman's *r* correlation; Figure 6).

**Table 3:** **Antifungal activity against the most abundant fungal species**.

**^#^Species (Number of tested)**	**Antifungal**	**MIC (μg/ml)**		^*^CBPs		
		**MIC_50_**	**MIC_90_**	**%S**	**%SDD**	**%R**
*Candida albicans (123)*	Fluconazole	0.5	>64	65.6	0.8	33.4
	Itraconazole	2	>8	29.3	5.7	65
	5-Flucytosine	0.125	0.5	98.4	0.8	0.8
*Candida lusitaniae (6)*	Fluconazole	0.125	0.5	100	0	0
	Itraconazole	0.0156	0.125	100	0	0
	5-Flucytosine	0.125	0.125	100	0	0
*Candida parapsilosis (40)*	Fluconazole	0.5	2	92.5	0	7.5
	Itraconazole	0.031	>8	75	5	20
	5-Flucytosine	0.125	0.125	100	0	0
Penicillium brevicompactum^*^ (13)	Fluconazole	0.125	0.125	100	0	0
	Itraconazole	0.0156	0.0156	92.5	0	7.5
	5-Flucytosine	0.125	0.125	100	0	0
Pichia fermentans^*^ (15)	Fluconazole	32	>64	15.4	0	84.6
	Itraconazole	0.25	4	44.7	20	33.3
	5-Flucytosine	0.5	2	92.3	7.7	0
Pichia kluyveri^*^ (9)	Fluconazole	32	32	11.1	0	88.9
	Itraconazole	0.125	0.125	88.9	11.1	0
	5-Flucytosine	0.5	0.5	100	0	0
Pichia manshurica^*^ (9)	Fluconazole	0.25	>64	77.8	0	22.2
	Itraconazole	0.0156	>8	77.8	0	22.2
	5-Flucytosine	0.125	8	77.8	11.1	11.1
Rhodotorula mucilaginosa^*^ (41)	Fluconazole	0.5	>64	63.4	0	36.6
	Itraconazole	0.0156	2	75.6	2.4	22
	5-Flucytosine	0.125	0.125	100	0	0
Torulaspora delbrueckii^*^ (23)	Fluconazole	0.125	8	87	0	13
	Itraconazole	0.0156	2	69.6	4.3	26.1
	5-Flucytosine	0.125	0.125	100	0	0

* species-specific CBPs are available only for Candida and Aspergillus spp.; for those non-Candida and non-Asperigillus isolates Candida and Asperigillus' CBPs have been used as a proxy;

# MIC_50_, MIC_90_, and CBPs have been calculated only for those species with number of isolates >5; S, sensible; SDD, Sensibility Dose-Dependent or Intermediate; R, resistant. MIC ranges: Fluconazole 0.125–64 μg/ml; Itraconazole 0.0156–8 μg/ml; 5-Flucytosine 0.125–64 μg/ml.

## Discussion

The vast majority of fungal species inhabiting our body are commensals and opportunistic pathogens that could turn into potential threats depending on strain virulence traits and on the status of the host's immune system. In this perspective to discover a pathogenic infection it seems crucial to define exactly which species are normally present in a given body district.

The human GI tract is known to contain variable communities of bacteria but also fungi have an important role in this ecological niche (Underhill and Iliev, 2014). Nevertheless, the phylogenetic characterization of fungal microorganisms and their specific role as part of the GI niche have not yet been studied extensively.

The advent of sanitation and food globalization has reduced the possibility for humans to come across with the richness of fungal species present in traditional fermented foods. Fungal infections are an ever increasing problem either as side effects of antibiotics use, high dose chemotherapy, and of the spread of immunosuppressive diseases. Estimates of global mortality rates suggest that fungi are responsible for more deaths than either tuberculosis or malaria (Brown et al., 2012). Most of this mortality is caused by species belonging to four fungal genera: *Aspergillus, Candida, Cryptococcus*, and *Pneumocystis* that are rapidly becoming resistant to most antifungal drugs (Brown et al., 2012; Denning and Bromley, 2015). The information on these fungi so far derives from the study of lung infections, while little is known on the gut mycobiota composition and its role in health and disease. The knowledge on the gut mycobiota is currently limited to few studies making it difficult to assess the significance of differences found in the intestinal fungal populations of diseases such as IBDs due to the lack of information on what the healthy mycobiota is. Here we aimed at defining the “healthy” gut mycobiota, showing that the intestinal fungal community of a cohort of Italian healthy volunteers is a variegate ecosystem that differs in function of individuals' life stage in a gender-dependent manner. We identified 34 fungal species of different ecological origins. While the majority of our fungal isolates has been previously described as inhabitants of the mammalian GI tract (Kurtzman et al., 2011; Rizzetto et al., 2014), some of the isolates belong to species so far identified in environmental samples only. Environmental fungi, in particular putative food-borne fungi, have been previously observed to be able to survive the transition through the GI tract possibly being metabolically active in the gut (David et al., 2014). The phenotypic properties of fungi isolated in this study suggested that these isolates are able to survive in the human GI tract, prompting the hypothesis of an ecological selection and potential ability to colonize this niche (David et al., 2014). Indeed the phenotypic features of the fungal isolates identified endow such isolates with an excellent ecological fitness in the human GI tract. We observed that approximately half of the isolates form hyphae or pseudohyphae, which are known to be involved in the adhesion to or penetration within the GI mucosa (Staab et al., 2013), consolidation of the colony, nutrient intake and formation of 3-dimensional matrices (Brand, 2011). A key factor of *C. albicans* commensalism/pathogenicity is its ability to switch between different morphologies, comprising cellular, pseudohyphae, and hyphae forms. As reported for *C. albicans*, the reversible transition to filamentous growth as a response to environmental cues (Sudbery, 2011) and phenotypic switching is essential for mucosal fungal colonization (Vautier et al., 2015).

Previous studies have also shown that *C. albicans* over-expresses a wide range of genes involved in resistance to high temperature and pH, oxidative stress, and hyphae formation during ileum and colon commensal colonization of BALB/c mice (Pierce et al., 2013). Similarly, the fungal isolates of this study, showing resistance to oxidative, high temperature, bile acids, and pH stresses may hold the potential to colonize the human gut. It is plausible that fecal fungal isolates with specific characteristics (such as high resistance to acidic pH and bile salts) survived to the gut environment, and that these traits make them able to colonize the gut. Thus, we can hypothesize a long process of evolution, selection or adaptation of environmental and food-borne strains to the human host, suggesting that pathogenic strains of commensal species can evolve through a repeated process of evolution and selection, depending on the immune status of the host (De Filippo et al., 2014). These findings encourage for in-depth, strain-level extensive studies on human gut mycobiota and the integration of such data with immunology to further establish the relevance of fungi in host physiology and host-microbe interaction. Furthermore, fungi may train host's immune system simply when passengers, rather than necessarily persisting only as continuous colonizers (Rizzetto et al., 2016).

We discovered that several fungal isolates displayed different levels of antifungal resistance. About 20 years ago, azole-sensitive *C. albicans* dominated infections, with other *Candida* species rarely observed. Actually *C. glabrata* is the second most-commonly isolated *Candida* species in the European Union and United States and has high rates of antifungal resistance (Slavin et al., 2015). Inappropriate antifungal use has contributed to the increase in antifungal resistance, causing objective complications for the treatment of invasive fungal infections that nowadays represent a severe cause of morbidity and mortality among immunocompromised individuals, neonates and elderly (Brown et al., 2012). Recent studies indicated that fungal infections may originate from individual's own commensal strains suggesting that the ability of a commensal microorganism to promote disease is not merely a consequence of impaired host immunity (Odds et al., 2006), suggesting that rural and other commercial uses of azole could be the culprit for the emergence of these resistant strains (Snelders et al., 2012). This underlines the risk that the increase of antifungal usage outside of the clinic could also lead to increased resistance to antifungals of individual's own commensal strains representing an important epidemiological problem in the future and remarking the importance to increase the investment in antifungal research.

It should be noted that all the samples analyzed by metagenomics resulted in high quality fungal sequences, indicating that all the fecal samples studied had fungal DNA. So far, the estimated ratio fungi/bacteria of 1:10000 (Huffnagle and Noverr, 2013), discourages an approach based on whole metagenome shotgun sequencing (Underhill and Iliev, 2014). We thus performed amplicon-based ITS1 metagenomics on a subset of healthy donors identifying more than 90 different fungal taxa. The first striking evidence was that metagenomics detected also sequences belonging to Agaricomycetes, among which several edible fungi, thus suggesting that dietary fungal intake is a potential confounding effect when studying the gut mycobiota. On the contrary 34 different fungal species were isolated using the culture-based approach. Both methods detected in any case differences in the diverse groups of study (Supplementary Figure S1). The discrepancies observed between culture-dependent and culture-independent approaches on the description of fungal populations could be attributed to the methodological differences of the two procedures applied suggesting that several of the fungal taxa identified by the metagenomics approach are not cultivable, either because we lack the proper culture conditions or because these belong to DNA from dead cells, environmental or food-borne fungi that cannot survive the passage through the GI tract, but whose DNA is still detectable. Furthermore, the DNA extraction method used in this study could not be suited to extract all the fungal DNA from the stool samples since the rare taxa *Yarrowia, Starmerella, Rhodosporidium*, and *Pleurostomophora* have been found only by the culture-based approach. On the other hand the culture condition that we used might be responsible for some of the discrepancies observed between the two methods. In our experience most of the commensal fungi commonly found in the human gut can be cultivated in YPD, yet other fungi that we were not able to cultivate might need different culture conditions from those we used in this work.

Although, for example, *S. cerevisiae* is often found in fermented food, it has been shown that it can survive GI tract challenges being a commensal of the human GI tract (Rizzetto et al., 2014) educating also adaptive immunity (Rizzetto et al., 2016). *S. cerevisiae* has been introduced in the human intestine through diet and fermented beverages and it has accompanied human evolution for at least the past 5150 years (Cavalieri et al., 2003). Our evidence, together with previous results, including a recent description of *S. cerevisiae* in IBDs (Sokol et al., 2016) showed that this microorganism is a potential commensal of the human intestine. The overall reduction of the amount and diversity of fungi introduced through consumption of fermented beverages suggests that the human gut mycobiota could be in dynamic change and certain potentially beneficial species could be lost as a result of modern food processing procedures, cultural changes, and food globalization. Ongoing studies on microbial anthropology in human populations consuming traditional fermented foods, hold the promise to shed light on the evolution of the fungal microbiota as associated to the evolution of diet. On the contrary the edible fungi belonging to Agaricomycetes cannot settle in the human gut due to their ecology (Hibbett, 2006) so we filtered-out these sequences for downstream analyses to reduce statistical noises on ecological measures, improving our results on the characterization of intestinal fungal communities. We are aware that other taxa identified by our analyses having environmental and food-borne origin may not be able to settle in the human gut, however little is known about these taxa while the Agaricomycetes sequences that we retrieved had a very low prevalence in the dataset and mostly belonged to edible fungi such as *Boletus, Suillus or Agrocybe*.

We further observed that amplicon-based ITS1 metagenomics cannot confidently describe fungal populations at a deeper level than genus overlooking species level information provided by the fungal cultivation approach (see Figures 1B, 2A). On the other hand, metagenomics analysis detected community structure differences that fungal cultivation did not identified (see Figures 2B–D). Nevertheless, the analysis of *alpha*-diversity from cultivation data on the subset of subjects used for the metagenomics analysis revealed no significant differences among genders remarking that the different sample sizes used in this work are an additional factor in the discrepancies observed between the two methods. Although the major limitation of culture-based methods for the study of microbial communities is the loss of ecological information due to the inability to cultivate most microorganisms by standard culturing techniques, fungi included, culture-based analysis of the human gut mycobiota is fundamental to discern fungal phenotypes that would be otherwise lost by metagenomics.

However, population level analyses with both approaches revealed interesting cues. As occurs for the bacterial microbiota, the intestinal mycobiota is shaped by host's age, gender, diet, and geographical environment (Yatsunenko et al., 2012; Hoffmann et al., 2013; David et al., 2014). Previous studies have shown that the development of the gut bacterial microbiota starts at birth with colonization by a low number of species from the vaginal and fecal microbiota of the mother and is characterized by many shifts in composition during infancy (Yatsunenko et al., 2012). Similarly, the mycobiota may show the same fate, but we observed an inverted trend in which the richness of the gut mycobiota of infants (0–2 years old) and children (3–10 years old) was higher than adults (≥18 years old). It has been shown that suppression of the bacterial microbiota upon treatment with antibiotics results in the outgrowth of the gut mycobiota (Dollive et al., 2013) probably as a consequence of reduced ecological competition. Similarly, a weak bacterial competition, in particular during infancy when the bacterial microbiota is less stable (Koenig et al., 2011; Lozupone et al., 2012), could be the reason why we observed an increased fungal *alpha*-diversity during the early stages of life or this could be due to the different interactions between intestinal fungi and diet (Hoffmann et al., 2013; David et al., 2014) which is peculiar during infancy. We also found that female subjects had a higher number of fungal isolates and different fungal species compared to male subjects and that female mycobiota cluster apart from male mycobiota. This may be ascribed to the role of sex hormones in modulating microbiota composition (Markle et al., 2013) and of diet in shifting the microbiota composition in a gender-dependent manner (Bolnick et al., 2014). Furthermore, the higher relative abundance of *Candida* in the fecal samples from female than male subjects could be also attributed to the prevalence of *Candida* species in the vaginal mycobiota (Drell et al., 2013) due to the anatomical proximity of the two districts. To the best of our knowledge, this is the first time that gender-related differences are described in the human gut mycobiota.

In conclusion we can state that culture-independent approaches are very promising for future investigation of the mycobiota, but yet require significant improvements in the selection of markers for amplicon-based metagenomics and the reference databases. Additionally development of markers targeting pathogenicity traits, including the genes involved in host invasion or evasion of immune defenses, or markers detecting resistance to azoles or other antifungals, is required to thoughtfully apply metagenomics to fungal infections, discriminating the healthy mycobiota from an altered one. Such improvement can be achieved only through systematic sequencing efforts of the cultivable mycobiota, paralleling what happened for the prokaryotic microbiota. In our experience, currently, the combination of the two methods compensated the methodological limits intrinsic in both approaches avoiding to overlook significant differences present in the gut mycobiota of healthy subjects.

## Availability of supporting data

Raw sequences are available in the European Nucleotide Archive (ENA) with accession number PRJEB11827 (http://www.ebi.ac.uk/ena/data/view/PRJEB11827).

## Author contributions

FS designed and performed the experiments, analyzed the data, and wrote the manuscript. IS and MD performed the experiments. IS, DA, and CD supervised and contributed to data analysis. PL and AC recruited subjects and collected specimens. IS, MD, LR, OJ, and CD critically reviewed the manuscript. DC and CDF conceived the study and approved the manuscript.

## Funding

This work was supported by the Accordo di Programma Integrato “MetaFoodLabs” funded by the research office of the Provincia Autonoma di Trento (Italy; PAT Prot. S216/2012/537723).

### Conflict of interest statement

The authors declare that the research was conducted in the absence of any commercial or financial relationships that could be construed as a potential conflict of interest.

## Supplementary material

Supplementary Table S1 **Correspondences between deposited metagenomics data and samples**.

Supplementary Table S2 **Phenotypic characteristics and antifungals susceptibility of fungal isolates**. ^*^calculated as the deviation of the inhibition halo diameter (Ø) from that of the M28-4D *S. cerevisiae* strain, according to the following formula: (Ø sample—Ø M284D strain)/Ø M284D strain ^*^100. ^#^0, non-invasive; 1, poor invasive; 2, invasive; 3, very invasive. −, no growth as measured by OD_630_ ≤ 0.2 or cfu/ml ≤ 10^5^; +, poor growth as measured by 0.2 < OD_630_ ≤ 0.7 or 10^5^ < cfu/ml ≤ 10^6^; ++ good growth as measured by 0.7 < OD_630_ ≤ 1.2 or 10^6^ < cfu/ml ≤ 10^7^; +++, very good growth as measured by OD_630_ > 1.2 or cfu/ml > 10^7^. na, not applicable; nd, not detected.

Supplementary Table S3 **Permutational multivariate analysis of variance (PERMANOVA) tests on unweighted and weighted UniFrac distances and Bray-Curtis dissimilarity**.

Supplementary Table S4 **Mean relative abundance (%) of OTUs at the genus level of fungal gut microbiota of healthy subjects from metagenomics analysis**.

Supplementary Figure S1 **Mean relative abundances of the gut mycobiota in the different groups of study measured according to (A) the culture-based approach (at species level) and (B) the amplicon-based ITS1 metagenomics approach (at genus level)**. In panel **(B)** are shown the most abundant genera (with relative abundances >0.1%) while all the other less abundant genera were grouped together and labeled as “*others*”.
