# Supplementary material for: Age and Gender Affect the Composition of Fungal Population of the Human Gastrointestinal Tract
Source: Front Microbiol. 2016 Aug 3;7:1227. doi: 10.3389/fmicb.2016.01227 (PMC4971113; doi:10.3389/fmicb.2016.01227)
Supplement: Supplementary Table S1 — Correspondences between deposited metagenomics data and samples. [file Table1.PDF]

Table S1: Correspondences between deposited metagenomics data and samples

Study_accession	Secondary_accession	Sample_accession	Experiment_accession	Run_accession	Sample_unique_name	File_name	Age	Gender	Age_group
PRJEB11827	SAMEA3670898	ERS978047	ERX1221136	ERR1142295	HS10	healthy_subject_10.fastq	2	Female	Infants
PRJEB11827	SAMEA3670899	ERS978048	ERX1221137	ERR1142296	HS13	healthy_subject_13.fastq	18	Female	Adults
PRJEB11827	SAMEA3670900	ERS978049	ERX1221138	ERR1142297	HS15	healthy_subject_15.fastq	11	Female	Adolescents
PRJEB11827	SAMEA3670901	ERS978050	ERX1221139	ERR1142298	HS20	healthy_subject_20.fastq	4	Female	Children
PRJEB11827	SAMEA3670902	ERS978051	ERX1221140	ERR1142299	HS21	healthy_subject_21.fastq	5	Female	Children
PRJEB11827	SAMEA3670903	ERS978052	ERX1221141	ERR1142300	HS22	healthy_subject_22.fastq	15	Female	Adolescents
PRJEB11827	SAMEA3670904	ERS978053	ERX1221142	ERR1142301	HS23	healthy_subject_23.fastq	11	Female	Adolescents
PRJEB11827	SAMEA3670905	ERS978054	ERX1221143	ERR1142302	HS27	healthy_subject_27.fastq	9	Female	Children
PRJEB11827	SAMEA3670906	ERS978055	ERX1221144	ERR1142303	HS29	healthy_subject_29.fastq	16	Female	Adolescents
PRJEB11827	SAMEA3670907	ERS978056	ERX1221145	ERR1142304	HS30	healthy_subject_30.fastq	12	Female	Adolescents
PRJEB11827	SAMEA3670908	ERS978057	ERX1221146	ERR1142305	HS31	healthy_subject_31.fastq	24	Female	Adults
PRJEB11827	SAMEA3670909	ERS978058	ERX1221147	ERR1142306	HS32	healthy_subject_32.fastq	32	Female	Adults
PRJEB11827	SAMEA3670910	ERS978059	ERX1221148	ERR1142307	HS33	healthy_subject_33.fastq	32	Female	Adults
PRJEB11827	SAMEA3670911	ERS978060	ERX1221149	ERR1142308	HS34	healthy_subject_34.fastq	25	Female	Adults
PRJEB11827	SAMEA3670912	ERS978061	ERX1221150	ERR1142309	HS35	healthy_subject_35.fastq	26	Female	Adults
PRJEB11827	SAMEA3670913	ERS978062	ERX1221151	ERR1142310	HS36	healthy_subject_36.fastq	20	Male	Adults
PRJEB11827	SAMEA3670914	ERS978063	ERX1221152	ERR1142311	HS37	healthy_subject_37.fastq	28	Female	Adults
PRJEB11827	SAMEA3670915	ERS978064	ERX1221153	ERR1142312	HS38	healthy_subject_38.fastq	25	Female	Adults
PRJEB11827	SAMEA3670916	ERS978065	ERX1221154	ERR1142313	HS39	healthy_subject_39.fastq	27	Female	Adults
PRJEB11827	SAMEA3670917	ERS978066	ERX1221155	ERR1142314	HS40	healthy_subject_40.fastq	27	Male	Adults
PRJEB11827	SAMEA3670918	ERS978067	ERX1221156	ERR1142315	HS41	healthy_subject_41.fastq	24	Female	Adults
PRJEB11827	SAMEA3670919	ERS978068	ERX1221157	ERR1142316	HS42	healthy_subject_42.fastq	24	Female	Adults
PRJEB11827	SAMEA3670920	ERS978069	ERX1221158	ERR1142317	HS43	healthy_subject_43.fastq	26	Male	Adults
PRJEB11827	SAMEA3670921	ERS978070	ERX1221159	ERR1142318	HS44	healthy_subject_44.fastq	24	Female	Adults
PRJEB11827	SAMEA3670922	ERS978071	ERX1221160	ERR1142319	HS45	healthy_subject_45.fastq	6	Female	Children
PRJEB11827	SAMEA3670923	ERS978072	ERX1221161	ERR1142320	HS46	healthy_subject_46.fastq	6	Female	Children
PRJEB11827	SAMEA3670924	ERS978073	ERX1221162	ERR1142321	HS47	healthy_subject_47.fastq	10	Female	Children

PRJEB11827	SAMEA3670925	ERS978074	ERX1221163	ERR1142322	HS48	healthy_subject_48.fastq	2.5	Female	Children
PRJEB11827	SAMEA3670926	ERS978075	ERX1221164	ERR1142323	HS49	healthy_subject_49.fastq	2.5	Male	Children
PRJEB11827	SAMEA3670927	ERS978076	ERX1221165	ERR1142324	HS50	healthy_subject_50.fastq	1.5	Female	Infants
PRJEB11827	SAMEA3670928	ERS978077	ERX1221166	ERR1142325	HS51	healthy_subject_51.fastq	8	Female	Children
PRJEB11827	SAMEA3670929	ERS978078	ERX1221167	ERR1142326	HS52	healthy_subject_52.fastq	23	Female	Adults
PRJEB11827	SAMEA3670930	ERS978079	ERX1221168	ERR1142327	HS53	healthy_subject_53.fastq	23	Female	Adults
PRJEB11827	SAMEA3670931	ERS978080	ERX1221169	ERR1142328	HS55	healthy_subject_55.fastq	2	Male	Infants
PRJEB11827	SAMEA3670932	ERS978081	ERX1221170	ERR1142329	HS56	healthy_subject_56.fastq	2	Male	Infants
PRJEB11827	SAMEA3670933	ERS978082	ERX1221171	ERR1142330	HS59	healthy_subject_59.fastq	5	Male	Children
PRJEB11827	SAMEA3670934	ERS978083	ERX1221172	ERR1142331	HS61	healthy_subject_61.fastq	2	Male	Infants
PRJEB11827	SAMEA3670935	ERS978084	ERX1221173	ERR1142332	HS63	healthy_subject_63.fastq	5	Male	Children
PRJEB11827	SAMEA3670936	ERS978085	ERX1221174	ERR1142333	HS65	healthy_subject_65.fastq	6	Male	Children
PRJEB11827	SAMEA3670937	ERS978086	ERX1221175	ERR1142334	HS66	healthy_subject_66.fastq	0.1	Male	Infants
PRJEB11827	SAMEA3670938	ERS978087	ERX1221176	ERR1142335	HS69	healthy_subject_69.fastq	6	Male	Children
PRJEB11827	SAMEA3670939	ERS978088	ERX1221177	ERR1142336	HS71	healthy_subject_71.fastq	1	Male	Infants
PRJEB11827	SAMEA3670940	ERS978089	ERX1221178	ERR1142337	HS74	healthy_subject_74.fastq	6	Male	Children
PRJEB11827	SAMEA3670941	ERS978090	ERX1221179	ERR1142338	HS76	healthy_subject_76.fastq	1	Male	Infants
PRJEB11827	SAMEA3670942	ERS978091	ERX1221180	ERR1142339	HS78	healthy_subject_78.fastq	12	Male	Adolescents
PRJEB11827	SAMEA3670943	ERS978092	ERX1221181	ERR1142340	HS79	healthy_subject_79.fastq	0.1	Male	Infants
PRJEB11827	SAMEA3670944	ERS978093	ERX1221182	ERR1142341	HS82	healthy_subject_82.fastq	10	Male	Children
PRJEB11827	SAMEA3670945	ERS978094	ERX1221183	ERR1142342	HS83	healthy_subject_83.fastq	12	Male	Adolescents
PRJEB11827	SAMEA3670946	ERS978095	ERX1221184	ERR1142343	HS86	healthy_subject_86.fastq	7	Male	Children
PRJEB11827	SAMEA3670947	ERS978096	ERX1221185	ERR1142344	HS87	healthy_subject_87.fastq	9	Male	Children
PRJEB11827	SAMEA3670948	ERS978097	ERX1221186	ERR1142345	HS88	healthy_subject_88.fastq	7	Male	Children
PRJEB11827	SAMEA3670949	ERS978098	ERX1221187	ERR1142346	HS89	healthy_subject_89.fastq	12	Male	Adolescents
PRJEB11827	SAMEA3670950	ERS978099	ERX1221188	ERR1142347	HS97	healthy_subject_97.fastq	6	Male	Children
PRJEB11827	SAMEA3670951	ERS978100	ERX1221189	ERR1142348	HS100	healthy_subject_100.fastq	0.1	Male	Infants
PRJEB11827	SAMEA3670952	ERS978101	ERX1221190	ERR1142349	HS101	healthy_subject_101.fastq	4	Male	Children
PRJEB11827	SAMEA3670953	ERS978102	ERX1221191	ERR1142350	HS103	healthy_subject_103.fastq	7	Male	Children
PRJEB11827	SAMEA3670954	ERS978103	ERX1221192	ERR1142351	HS104	healthy_subject_104.fastq	4	Male	Children
