# Supplementary material for: Age and Gender Affect the Composition of Fungal Population of the Human Gastrointestinal Tract
Source: Front Microbiol. 2016 Aug 3;7:1227. doi: 10.3389/fmicb.2016.01227 (PMC4971113; doi:10.3389/fmicb.2016.01227)
Supplement: Supplementary Table S2 — Phenotypic characteristics and antifungals susceptibility of fungal isolates. *calculated as the deviation of the inhibition halo diameter (Ø) from that of the M28-4D S. cerevisiae strain, according to the following formula: (Ø sample—Ø M284D strain)/Ø M284D strain *100. #0, non-invasive; 1, poor invasive; 2, invasive; 3, very invasive. −, no growth as measured by OD630 ≤ 0.2 or cfu/ml ≤ 105; +, poor growth as measured by 0.2 < OD630 ≤ 0.7 or 105 < cfu/ml ≤ 106; ++ good growth as measured by 0.7 < OD630 ≤ 1.2 or 106 < cfu/ml ≤ 107; +++, very good growth as measured by OD630 > 1.2 or cfu/ml > 107. na, not applicable; nd, not detected. [file Table2.PDF]

Table S2: Phenotypic characteristics and antifungals susceptibility of fungal isolates.

Isolate ID	Subject	Sex	Age	Fluconazole (MIC µg/ml)	5-Flucytosine (MIC µg/ml)	Itraconazole (MIC µg/ml)	pH2	pH3	oxbile 0.5%	oxbile 1%	oxbile 2%	*Ox stress resistance	# Agar invasivity	Hyphae and pseudohyphae	37°C	40°C	42°C	44°C	46°C	*Species*
YHS1	HS1	M	5	na	na	na	na	na	na	na	na	na	na	na	na	na	na	na	na	*Candida albicans*
YHS2	HS2	M	5	0. 25	0. 125	0. 0156	-	-	-	-	-	-33.3	0	nd	++	+	-	+	-	*Saccharomyces cerevisiae*
YHS3	HS3	M	14	na	na	na	na	na	na	na	na	na	na	na	na	na	na	na	na	*Candida albicans*
YHS4	HS4	M	1	64	0. 125	0. 5	-	++	+++	+++	+++	-26.7	na	na	++	++	-	-	-	*Candida albicans*
YHS5	HS4	M	1	na	na	na	na	na	na	na	na	na	na	na	na	na	na	na	na	*Yarrowia lipolytica*
YHS6	HS7	F	20	> 64	0. 125	> 8	++	+++	+++	+++	+++	-66.7	1	nd	+++	+++	+++	+++	+	*Saccharomyces cerevisiae*
YHS7	HS8	M	5	64	0. 125	1	-	++	+++	+++	+++	-20	0	nd	++	++	+	-	-	*Saccharomyces cerevisiae*
YHS8	HS9	M	14	na	na	na	na	na	na	na	na	na	na	na	na	na	na	na	na	*Candida parapsilosis*
YHS9	HS10	F	2	na	na	na	na	na	na	na	na	na	na	na	na	na	na	na	na	*Candida albicans*
YHS10	HS11	M	16	na	na	na	na	na	na	na	na	na	na	na	na	na	na	na	na	*Candida albicans*
YHS11	HS12	M	15	na	na	na	na	na	na	na	na	na	na	na	na	na	na	na	na	*Candida albicans*
YHS12	HS13	F	18	na	na	na	na	na	na	na	na	na	na	na	na	na	na	na	na	*Pichia carribica*
YHS13	HS14	F	0.3	0. 125	0. 25	0. 25	+	+++	++	++	++	-40	0	nd	+++	++	++	-	-	*Pichia fermentans*
YHS14	HS14	F	0.3	0. 125	0. 5	0. 125	+	+++	+++	+++	++	-26.67	0	nd	+++	++	+	-	-	*Pichia kluyveri*
YHS15	HS14	F	0.3	32	0. 5	0. 031	-	+++	+++	+++	++	-26.67	1	nd	+++	+	-	-	-	*Pichia fermentans*
YHS16	HS14	F	0.3	32	1	1	+	++	+++	+++	+++	-33.33	1	nd	+++	+	-	-	-	*Pichia fermentans*
YHS17	HS14	F	0.3	> 64	4	4	+	++	++	++	++	-26.67	0	nd	+++	+	-	-	-	*Pichia fermentans*
YHS18	HS14	F	0.3	> 64	2	4	+	+++	++	++	++	-26.67	1	nd	+++	++	-	-	-	*Pichia fermentans*
YHS19	HS14	F	0.3	32	0. 5	0. 125	+	+++	+++	++	++	-26.67	1	nd	+++	++	-	-	-	*Pichia fermentans*
YHS20	HS14	F	0.3	32	0. 5	0. 25	-	+++	+++	++	++	-26.67	1	nd	+++	++	-	-	-	*Pichia fermentans*
YHS21	HS14	F	0.3	> 64	1	4	+	+++	+++	++	++	-33.33	1	nd	+++	+	-	-	-	*Pichia fermentans*
YHS22	HS14	F	0.3	32	0. 5	0. 25	+	+++	+++	++	++	-33.33	1	nd	+++	+	-	-	-	*Pichia fermentans*
YHS23	HS14	F	0.3	32	0. 5	0. 125	++	+++	+++	++	++	-26.67	1	nd	+++	+	-	-	-	*Pichia fermentans*
YHS24	HS14	F	0.3	> 64	2	> 8	-	++	+++	++	++	-26.67	1	nd	+++	+++	-	-	-	*Pichia fermentans*
YHS25	HS14	F	0.3	32	0. 5	0. 125	-	+++	+++	++	++	-33.33	0	nd	+++	+	+	-	-	*Pichia fermentans*
YHS26	HS14	F	0.3	32	0. 5	0. 125	-	+++	+++	++	++	-33.33	0	nd	+++	+	-	-	-	*Pichia fermentans*
YHS27	HS14	F	0.3	32	0. 5	0. 125	-	+++	+++	++	++	-26.67	0	nd	+++	+	-	-	-	*Pichia fermentans*
YHS28	HS14	F	0.3	32	0. 5	0. 125	-	+++	+++	++	++	-26.67	0	nd	+++	+	-	-	-	*Pichia kluyveri*
YHS29	HS14	F	0.3	32	0. 5	0. 125	-	+++	+++	++	++	-26.67	0	nd	+++	+	-	-	-	*Pichia kluyveri*
YHS30	HS14	F	0.3	32	0. 5	0. 125	-	+++	+++	+++	++	-26.67	0	nd	+++	+	-	-	-	*Pichia kluyveri*
YHS31	HS14	F	0.3	32	0. 5	0. 125	-	+++	+++	++	++	-26.67	0	nd	+++	+	-	-	-	*Pichia kluyveri*
YHS32	HS14	F	0.3	32	0. 5	0. 25	-	+++	+++	++	++	-26.67	0	nd	+	+	-	-	-	*Pichia kluyveri*

YHS33	HS14	F	0.3	32	0. 5	0. 125	-	+++	+++	++	++	-33.33	0	nd	+++	-	-	-	-	*Pichia kluyveri*
YHS34	HS14	F	0.3	32	0. 5	0. 125	-	+++	+++	++	++	-26.67	0	nd	+++	+	-	-	-	*Pichia kluyveri*
YHS35	HS14	F	0.3	32	0. 5	0. 125	-	+++	+++	++	++	-26.67	0	nd	+++	+	-	-	-	*Pichia kluyveri*
YHS36	HS16	M	14	na	na	na	na	na	na	na	na	na	na	na	na	na	na	na	na	*Candida zelanoydes*
YHS37	HS17	M	15	na	na	na	na	na	na	na	na	na	na	na	na	na	na	na	na	*Candida tropicalis*
YHS38	HS18	M	11	na	na	na	na	na	na	na	na	na	na	na	na	na	na	na	na	*Candida albicans*
YHS39	HS19	F	3	na	na	na	na	na	na	na	na	na	na	na	na	na	na	na	na	*Candida lusitaniae*
YHS40	HS21	F	5	0. 5	0. 125	1	++	+++	+++	+++	+++	-9.09	2	nd	+++	+++	+++	++	-	*Torulaspora delbrueckii*
YHS41	HS22	F	15	1	0. 125	2	++	+++	+++	+++	+++	-18.18	2	nd	+++	+++	+++	++	-	*Candida albicans*
YHS42	HS22	F	15	0. 5	0. 125	0. 125	++	+++	+++	+++	+++	-18.18	2	Hyphae	+++	+++	+++	++	-	*Candida albicans*
YHS43	HS22	F	15	na	na	na	-	na	na	na	na	na	na	na	na	na	na	na	na	*Candida albicans*
YHS44	HS24	M	15	na	na	na	na	na	na	na	na	na	na	na	na	na	na	na	na	*Cryptococcus saitoi*
YHS45	HS25	M	7	na	na	na	-	na	na	na	na	na	na	na	na	na	na	na	na	*Candida albicans*
YHS46	HS26	M	3	na	na	na	na	na	na	na	na	na	na	na	na	na	na	na	na	*Candida lusitaniae*
YHS47	HS27	F	9	na	na	na	na	+++	na	na	na	-50	2	Pseudohyphae	+++	+++	+++	+++	-	*Candida albicans*
YHS48	HS28	M	5	na	na	na	na	na	na	na	na	na	na	na	na	na	na	na	na	*Rhodotorula mucilaginosa*
YHS49	HS29	F	16	0. 5	0. 125	0. 0156	-	+++	+++	+++	+++	-45.45	3	Pseudohyphae	+++	+++	+	+	-	*Candida parapsilosis*
YHS50	HS29	F	16	0. 125	0. 125	0. 0156	+	+++	+++	+++	+++	-36.36	3	Pseudohyphae	+++	+++	++	+	-	*Candida parapsilosis*
YHS51	HS29	F	16	0. 5	0. 125	0. 0156	+	+++	+++	+++	+++	-36.36	3	Pseudohyphae	+++	+++	+++	++	-	*Candida parapsilosis*
YHS52	HS29	F	16	0. 5	0. 125	0. 0156	++	+++	+++	+++	+++	-36.36	3	Pseudohyphae	+++	+++	+++	++	-	*Candida parapsilosis*
YHS53	HS29	F	16	0. 5	0. 125	0. 0156	-	+++	+++	+++	+++	-63.64	3	Pseudohyphae	+++	+++	++	+	-	*Candida parapsilosis*
YHS54	HS29	F	16	0. 5	0. 25	0. 0156	-	+++	+++	+++	+++	-63.64	3	Pseudohyphae	+++	+++	+	+	-	*Candida parapsilosis*
YHS55	HS29	F	16	0. 5	0. 125	1	++	+++	+++	+++	+++	-63.64	3	Pseudohyphae	+++	+++	+++	++	-	*Rhodotorula mucilaginosa*
YHS56	HS29	F	16	0. 25	0. 125	0. 0156	-	+++	+++	+++	+++	-45.45	3	Pseudohyphae	+++	+++	+++	++	-	*Rhodotorula mucilaginosa*
YHS57	HS29	F	16	na	na	na	na	na	na	na	na	na	na	na	na	na	na	na	na	*Pichia kluyveri*
YHS58	HS31	F	24	2	1	0. 0156	-	+++	+++	++	++	-46.7	3	Pseudohyphae	+++	+++	+++	+	-	*Candida parapsilosis*
YHS59	HS31	F	24	0. 5	4	0. 0156	-	+++	+++	+++	+++	-56.79	3	Hyphae	+++	+++	+++	++	-	*Candida albicans*
YHS60	HS31	F	24	na	na	na	na	+++	na	na	na	-100	3	Pseudohyphae	+++	-	-	-	-	*Candida parapsilosis*
YHS61	HS31	F	24	0. 25	64	0. 0156	+	+++	+++	+++	+++	-53.3	0	nd	+++	+++	+++	++	-	*Candida albicans*
YHS62	HS31	F	24	na	na	na	na	-	na	na	na	-100	0	nd	+++	+++	+++	-	-	*Candida albicans*
YHS63	HS31	F	24	na	na	na	na	-	na	na	na	-100	0	nd	+++	+++	+++	-	-	*Candida albicans*
YHS64	HS31	F	24	1	0. 125	0. 031	-	++	+	+	+	-62.96	0	nd	+++	+++	+++	-	-	*Rhodotorula mucilaginosa*
YHS65	HS31	F	24	na	na	na	na	+++	na	na	na	-100	0	nd	+++	+++	+++	-	-	*Candida lusitaniae*
YHS66	HS31	F	24	0. 5	0. 5	0. 0156	++	+++	+++	+++	+++	-40	0	nd	+++	+++	+++	+	+	*Rhodotorula mucilaginosa*
YHS67	HS31	F	24	0. 5	0. 125	0. 0156	-	+++	+++	+++	+++	-66.7	0	nd	+++	+	-	-	-	*Rhodosporidium kratochvilovae*
YHS68	HS31	F	24	na	na	na	na	-	na	na	na	-75.31	1	Hyphae	+++	-	-	-	-	*Candida albicans*

YHS69	HS31	F	24	na	na	na	na	-	na	na	na	-56.79	0	nd	+++	-	-	-	-	*Candida albicans*
YHS70	HS31	F	24	na	na	na	na	+++	na	na	na	-100	1	nd	+++	+++	+++	-	-	*Candida albicans*
YHS71	HS31	F	24	0. 125	0. 125	0. 0156	-	-	-	-	-	16.7	0	nd	+++	+++	+++	-	-	*Candida albicans*
YHS72	HS31	F	24	0. 25	0. 5	0. 0156	-	+++	+++	+++	++	-73.3	0	nd	+++	+++	+++	-	-	*Candida lusitaniae*
YHS73	HS31	F	24	0. 125	0. 125	0. 0156	-	+	-	-	-	6.7	1	nd	+++	+++	+++	-	-	*Candida albicans*
YHS74	HS31	F	24	na	na	na	na	-	na	na	na	-100	1	nd	+++	+++	+++	-	-	*Candida albicans*
YHS75	HS31	F	24	na	na	na	na	+++	na	na	na	-100	3	Pseudohyphae	+++	+++	+++	-	-	*Candida parapsilosis*
YHS76	HS31	F	24	na	na	na	na	-	na	na	na	-100	1	nd	+++	+++	+++	-	-	*Candida albicans*
YHS77	HS32	F	32	0. 125	0. 5	0. 0156	-	+++	+++	+++	+++	13.3	0	nd	+++	+++	+++	-	-	*Pichia manshurica*
YHS78	HS32	F	32	0. 25	> 64	0. 0156	-	+++	+++	+++	+++	16.7	2	nd	+++	+++	+++	++	-	*Pichia manshurica*
YHS79	HS32	F	32	na	na	na	na	+++	na	na	na	-75.31	2	Hyphae	+++	+++	+++	-	-	*Pichia manshurica*
YHS80	HS32	F	32	0. 25	8	0. 0156	+	+++	+++	+++	+++	-66.7	0	nd	+++	+++	+++	+	-	*Pichia manshurica*
YHS81	HS32	F	32	> 64	0. 125	> 8	++	+++	+++	+++	++	-33.3	0	nd	+++	+++	+++	++	-	*Pichia manshurica*
YHS82	HS32	F	32	na	na	na	na	+++	na	na	na	-50.62	0	nd	+++	+++	+++	-	-	*Pichia manshurica*
YHS83	HS32	F	32	0. 5	0. 5	0. 0156	-	+++	+++	+++	++	16.7	2	nd	+++	+++	+++	-	-	*Pichia manshurica*
YHS84	HS32	F	32	0. 25	0. 125	0. 0156	++	+++	+++	+++	+++	0	1	nd	+++	+++	+++	++	-	*Pichia manshurica*
YHS85	HS32	F	32	0. 25	0. 125	0. 0156	++	+++	+++	+++	+++	0	2	Pseudohyphae	+++	+++	+++	++	-	*Pichia manshurica*
YHS86	HS32	F	32	> 64	0. 125	> 8	+	+++	+++	+++	+++	-66.7	1	nd	+++	+++	+++	+++	+	*Pichia manshurica*
YHS87	HS32	F	32	na	na	na	na	+++	na	na	na	-81.48	2	nd	+++	+++	+++	-	-	*Pichia manshurica*
YHS88	HS33	F	32	na	na	na	na	+++	na	na	na	-44.44	0	nd	+++	+++	+++	-	-	*Rhodotorula mucilaginosa*
YHS89	HS33	F	32	> 64	0. 125	> 8	+	+++	+++	+++	+++	-26.7	1	nd	+++	+++	+++	++	-	*Candida albicans*
YHS90	HS33	F	32	> 64	0. 125	> 8	+	+++	+++	+++	+++	-66.7	3	Hyphae	+++	+++	+++	++	-	*Candida albicans*
YHS91	HS33	F	32	0. 25	0. 125	0. 0156	+	+++	+++	+++	+++	-20	3	Hyphae	+++	+++	+++	+	-	*Candida albicans*
YHS92	HS33	F	32	0. 5	0. 125	0. 0156	++	+++	+++	+++	+++	-20	3	Hyphae	+++	+++	+	+	-	*Candida albicans*
YHS93	HS33	F	32	0. 25	0. 125	0. 0156	++	+++	+++	+++	+++	-26.7	2	nd	+++	+++	+++	+	-	*Pichia manshurica*
YHS94	HS33	F	32	> 64	0. 125	> 8	+	+++	+++	+++	+++	-26.7	1	nd	+++	+++	+++	++	-	*Candida albicans*
YHS95	HS33	F	32	0. 25	0. 125	0. 0156	++	+++	+++	+++	+++	0	3	nd	+++	+++	+++	++	-	*Candida albicans*
YHS96	HS33	F	32	0. 25	0. 125	0. 0156	++	+++	+++	+++	+++	-13.3	3	Hyphae	+++	+++	+++	++	-	*Candida albicans*
YHS97	HS33	F	32	0. 25	0. 125	0. 0156	++	+++	+++	+++	+++	-50.62	3	Hyphae	+++	+++	+++	++	+	*Candida albicans*
YHS98	HS33	F	32	> 64	0. 125	> 8	++	+++	+++	+++	+++	-26.7	0	Hyphae	+++	+++	+	+	-	*Rhodosporidium kratochvilovae*
YHS99	HS34	F	25	> 64	0. 125	> 8	++	+++	+++	+++	+++	-66.7	3	nd	+++	+++	+++	+++	+	*Candida albicans*
YHS100	HS36	M	20	0. 125	0. 125	0. 0156	-	+++	-	-	-	-100	0	Hyphae	+++	+++	+++	+++	-	*Candida albicans*
YHS101	HS36	M	20	0. 125	0. 125	0. 0156	-	+++	+	-	-	-100	1	Hyphae	+++	+++	+++	+++	-	*Torulaspora delbrueckii*
YHS102	HS36	M	20	0. 125	0. 125	0. 0156	-	+++	+	-	-	-100	0	nd	+++	+++	+++	+++	-	*Candida albicans*
YHS103	HS36	M	20	0. 125	0. 125	0. 0156	-	+++	-	-	-	-100	0	nd	+++	+++	+++	+++	-	*Candida glabrata*
YHS104	HS36	M	20	0. 125	0. 125	2	+	+++	+++	+++	+++	-7.41	3	Hyphae	+++	+++	+++	+++	-	*Candida parapsilosis*

YHS105	HS36	M	20	> 64	0. 5	2	++	+++	+++	+++	+++	-100	3	Hyphae	+++	+++	+++	+++	-	*Starmerella bacillaris*
YHS106	HS37	F	28	0. 25	0. 5	1	+	+++	+++	+++	+++	4.94	3	Hyphae	+++	+++	+++	+++	-	*Candida albicans*
YHS107	HS37	F	28	0. 25	0. 5	2	++	+++	+++	+++	+++	4.94	1	Hyphae	+++	+++	+++	+++	-	*Candida albicans*
YHS108	HS37	F	28	0. 25	0. 5	2	++	+++	+++	+++	+++	23.46	1	Hyphae	+++	+++	+++	+++	-	*Candida albicans*
YHS109	HS37	F	28	0. 5	0. 5	2	++	+++	+++	+++	+++	17.28	1	Hyphae	+++	+++	+++	+++	-	*Torulaspora delbrueckii*
YHS110	HS37	F	28	0. 5	0. 5	2	++	+++	+++	+++	+++	17.28	2	Hyphae	+++	+++	+++	+++	-	*Candida albicans*
YHS111	HS38	F	25	0. 125	0. 125	0. 0156	-	+++	-	-	-	-33.33	1	nd	++	+	+	+	-	*Candida deformans*
YHS112	HS39	F	27	> 64	0. 125	> 8	+	+++	+++	+++	+++	-100	0	nd	+++	+++	+++	+++	-	*Candida albicans*
YHS113	HS39	F	27	> 64	0. 125	0. 125	+	+++	+++	+++	+++	-13.58	3	Hyphae	+++	+++	+++	+++	-	*Candida albicans*
YHS114	HS39	F	27	> 64	0. 125	> 8	+	+++	+++	+++	+++	-100	0	nd	+++	+++	+++	+++	-	*Candida albicans*
YHS115	HS39	F	27	> 64	0. 125	0. 125	+	+++	+++	+++	+++	-7.41	3	Hyphae	+++	+++	+++	+++	-	*Candida albicans*
YHS116	HS39	F	27	> 64	0. 125	> 8	+	+++	+++	+++	+++	-100	0	nd	+++	+++	+++	+++	-	*Candida albicans*
YHS117	HS39	F	27	32	0. 125	1	+	+++	+++	+++	+++	-13.58	1	nd	+++	+++	+++	+++	-	*Candida albicans*
YHS118	HS40	M	27	0. 125	0. 125	0. 0156	-	+++	+++	+++	+++	20	3	Hyphae	++	+	+	-	-	*Pennicillium crustosum*
YHS119	HS40	M	27	0. 125	0. 125	0. 0156	-	+++	+++	+++	+++	20	3	Hyphae	++	+	+	-	-	*Pennicillium crustosum*
YHS120	HS40	M	27	0. 125	0. 125	0. 0156	-	+++	+++	+++	+++	20	3	Hyphae	++	+	+	-	-	*Pennicillium crustosum*
YHS121	HS40	M	27	0. 125	0. 125	0. 0156	-	+++	+++	+++	+++	20	3	Hyphae	++	+	+	-	-	*Pennicillium crustosum*
YHS122	HS40	M	27	0. 125	0. 125	0. 0156	-	+++	+++	+++	+++	20	3	Hyphae	++	+	+	-	-	*Pennicillium crustosum*
YHS123	HS41	F	24	0. 25	0. 125	0. 0156	-	++	+++	+++	+++	-33.33	2	Hyphae	+++	+++	+	-	-	*Candida parapsilosis*
YHS124	HS41	F	24	1	0. 125	0. 0156	-	+++	+++	+++	+++	-53.33	2	Hyphae	+++	++	-	-	-	*Candida parapsilosis*
YHS125	HS41	F	24	> 64	0. 125	0. 0156	-	-	+	+	+	20	3	Hyphae	+	+	+	+	-	*Aspergillus glaucus*
YHS126	HS43	M	26	0. 5	0. 125	0. 0156	-	+++	+++	+++	+++	-100	2	nd	+++	-	-	-	-	*Candida parapsilosis*
YHS127	HS44	F	24	0. 125	0. 125	0. 125	-	+++	+++	+++	++	-7.41	1	Hyphae	+++	+++	+++	+++	-	*Candida albicans*
YHS128	HS44	F	24	0. 125	0. 125	0. 0156	-	+++	+++	+++	+++	-7.41	2	nd	+++	+++	+++	+++	-	*Candida albicans*
YHS129	HS44	F	24	0. 125	0. 125	0. 25	-	+++	+++	+++	+++	4.94	0	Hyphae	+++	+++	+++	+++	-	*Torulaspora delbrueckii*
YHS130	HS44	F	24	0. 5	0. 125	0. 25	+	+++	+++	+++	+++	-7.41	0	nd	+++	+++	+++	+++	-	*Candida albicans*
YHS131	HS44	F	24	0. 5	0. 125	0. 25	+	+++	-	-	-	-100	2	Hyphae	+++	+++	+++	+++	-	*Candida albicans*
YHS132	HS44	F	24	0. 125	0. 125	0. 0156	+	+++	+++	+++	+++	-7.41	3	Pseudohyphae	+++	+++	+++	+++	-	*Candida parapsilosis*
YHS133	HS44	F	24	> 64	0. 5	0. 125	+	+++	+++	+++	+++	-7.41	2	nd	+++	+++	+++	+++	-	*Candida parapsilosis*
YHS134	HS46	F	6	0. 5	0. 125	2	++	-	+++	+++	+++	-18.18	3	Hyphae	+++	+++	+++	++	-	*Candida albicans*
YHS135	HS46	F	6	0. 5	0. 125	2	++	+++	+++	+++	+++	-27.27	2	Hyphae	+++	+++	+++	++	-	*Candida albicans*
YHS136	HS46	F	6	0. 5	0. 125	2	++	+++	+++	+++	+++	-27.27	2	Hyphae	+++	+++	+++	++	-	*Candida albicans*
YHS137	HS46	F	6	0. 5	0. 125	2	+	+++	+++	+++	+++	-27.27	2	Hyphae	+++	+++	+++	++	-	*Candida albicans*
YHS138	HS47	F	10	0. 125	0. 125	0. 0156	-	+++	-	-	-	0	2	Hyphae	+++	+++	+++	++	-	*Torulaspora delbrueckii*
YHS139	HS47	F	10	0. 25	0. 125	1	++	+++	+++	+++	+++	-18.18	2	Hyphae	+++	+++	+++	++	-	*Candida albicans*
YHS140	HS47	F	10	0. 25	0. 125	0. 125	++	+++	+++	+++	+++	-54.55	2	Hyphae	+++	+++	+++	++	-	*Torulaspora delbrueckii*

YHS141	HS47	F	10	0. 25	0. 125	1	++	+++	+++	+++	+++	-9.09	3	Hyphae	+++	+++	+++	++	-	*Candida albicans*
YHS142	HS50	F	1.5	0. 125	0. 125	0. 0156	-	+++	+	-	-	27.27	2	nd	+++	+++	+++	++	-	*Candida intermedia*
YHS143	HS50	F	1.5	0. 125	0. 125	0. 0156	-	+++	+++	+++	+++	-18.18	1	nd	+++	+++	+++	++	-	*Candida lusitaniae*
YHS144	HS50	F	1.5	0. 5	0. 125	0. 0156	++	+++	+++	+++	+++	-18.18	1	Hyphae	+++	+++	+++	++	-	*Candida albicans*
YHS145	HS50	F	1.5	0. 25	0. 125	0. 0156	++	++	+++	+++	+++	63.64	1	Pseudohyphae	+++	++	+++	++	-	*Rhodotorula mucilaginosa*
YHS146	HS50	F	1.5	0. 25	0. 125	0. 0156	++	-	++	++	+	-18.18	1	nd	+++	+++	+++	+	-	*Candida lusitaniae*
YHS147	HS50	F	1.5	0. 5	0. 125	0. 125	-	+++	+++	+++	+++	-54.55	1	nd	+++	+++	+++	++	-	*Candida parapsilosis*
YHS148	HS50	F	1.5	0. 5	0. 125	0. 125	++	+++	+++	+++	+++	36.36	1	nd	+++	+++	+++	++	-	*Candida lusitaniae*
YHS149	HS50	F	1.5	0. 5	0. 125	0. 0156	-	+++	+++	+++	+++	-27.27	3	Pseudohyphae	+++	+++	+++	+	-	*Candida parapsilosis*
YHS150	HS50	F	1.5	na	na	na	na	na	na	na	na	na	na	na	na	na	na	na	na	*Yarrowia lipolytica*
YHS151	HS51	F	8	na	na	na	na	+++	na	na	na	36.36	1	nd	+++	+++	+++	++	-	*Rhodotorula mucilaginosa*
YHS152	HS52	F	23	0. 25	0. 125	0. 0156	-	+++	+++	+++	+++	-7.41	1	Hyphae	+++	+++	+++	+++	-	*Candida albicans*
YHS153	HS52	F	23	0. 25	0. 125	0. 0156	+	+++	+++	+++	+++	-7.41	1	Hyphae	+++	+++	+++	+++	-	*Candida albicans*
YHS154	HS52	F	23	0. 125	0. 125	0. 0156	++	+++	+++	+++	+++	4.94	1	Hyphae	+++	+++	+++	+++	-	*Candida albicans*
YHS155	HS52	F	23	0. 5	0. 125	0. 0156	-	+++	+++	+++	+++	-7.41	3	Hyphae	+++	+++	+++	+++	-	*Candida albicans*
YHS156	HS53	F	23	0. 5	0. 125	0. 0156	++	+++	+++	+++	+++	-27.27	2	Hyphae	+++	+++	+++	++	-	*Candida albicans*
YHS157	HS53	F	23	0. 5	0. 125	2	++	+++	+++	+++	+++	-18.18	1	nd	+++	+++	+++	++	-	*Candida albicans*
YHS158	HS54	M	2	0. 125	0. 125	0. 0156	-	+++	+++	+++	+++	-40	1	Pseudohyphae	+++	+++	+++	+++	+	*Candida albicans*
YHS159	HS54	M	2	0. 5	0. 125	> 8	+	+++	+++	+++	+++	-53.33	2	Hyphae	+++	+++	+	-	-	*Candida parapsilosis*
YHS160	HS54	M	2	0. 25	0. 125	1	+	+++	na	na	na	-43.33	2	Hyphae	++	+++	+	+	+	*Candida albicans*
YHS161	HS54	M	2	0. 25	0. 125	2	+	+++	+++	+++	+++	-46.67	1	nd	+++	+++	+	+	+	*Candida albicans*
YHS162	HS54	M	2	0. 5	0. 125	4	+	+++	+++	+++	+++	-33.33	1	nd	+++	+++	+++	+++	+	*Candida albicans*
YHS163	HS54	M	2	0. 5	0. 125	2	+	++	+++	+++	+++	-46.67	2	Hyphae	+++	+++	+++	++	+	*Candida albicans*
YHS164	HS54	M	2	0. 25	0. 125	2	+	na	++	++	++	-33.33	0	nd	na	na	na	na	na	*Lichtheimia ramosa*
YHS165	HS55	M	2	> 64	0. 125	0. 0156	-	+	+++	+++	+++	na	1	Hyphae	+++	++	-	-	-	*Aspergillus glaucus*
YHS166	HS56	M	2	0. 25	0. 5	2	+	+++	+++	+++	+++	-20	0	nd	+++	++	++	++	+	*Candida albicans*
YHS167	HS57	F	12	0. 125	0. 125	0. 0156	-	+	-	-	-	-26.67	3	Hyphae	++	++	-	-	-	*Penicillium brevicompactum*
YHS168	HS57	F	12	0. 125	0. 125	0. 0156	-	+	-	-	-	-26.67	3	Hyphae	++	++	-	-	-	*Penicillium brevicompactum*
YHS169	HS57	F	12	0. 125	0. 125	0. 0156	-	+	-	-	-	-26.67	3	Hyphae	++	++	-	-	-	*Penicillium brevicompactum*
YHS170	HS57	F	12	0. 125	0. 125	0. 0156	-	+	-	-	-	-26.67	3	Hyphae	++	++	-	-	-	*Penicillium brevicompactum*
YHS171	HS57	F	12	0. 125	0. 125	0. 0156	-	+	-	-	-	-26.67	3	Hyphae	++	++	-	-	-	*Penicillium brevicompactum*
YHS172	HS57	F	12	0. 125	0. 125	0. 0156	-	+	-	-	-	-26.67	3	Hyphae	++	++	-	-	-	*Penicillium brevicompactum*
YHS173	HS57	F	12	0. 125	0. 125	0. 0156	-	+	-	-	-	-26.67	3	Hyphae	++	++	-	-	-	*Penicillium brevicompactum*
YHS174	HS57	F	12	0. 125	0. 125	0. 0156	-	+	-	-	-	-26.67	3	Hyphae	++	++	-	-	-	*Penicillium brevicompactum*
YHS175	HS57	F	12	0. 125	0. 125	0. 0156	-	+	-	-	-	-26.67	3	Hyphae	++	++	-	-	-	*Penicillium brevicompactum*
YHS176	HS57	F	12	0. 125	0. 125	0. 0156	-	+	-	-	-	-26.67	3	Hyphae	++	++	-	-	-	*Penicillium brevicompactum*

YHS177	HS57	F	12	0. 125	0. 125	0. 0156	-	+	-	-	-	-26.67	3	Hyphae	++	++	-	-	-	*Penicillium brevicompactum*
YHS178	HS57	F	12	0. 125	0. 125	0. 0156	-	+	-	-	-	-26.67	3	Hyphae	++	++	-	-	-	*Penicillium brevicompactum*
YHS179	HS58	F	3	0. 125	0. 125	0. 0156	-	+++	+++	+++	+++	-33.33	0	nd	+++	+++	++	++	+	*Candida albicans*
YHS180	HS60	F	3	0. 5	0. 125	0. 0625	+	+++	+++	+++	+++	-23.33	1	nd	++	++	++	++	+	*Candida albicans*
YHS181	HS61	M	2	0. 5	0. 125	4	+	++	+++	++	++	-26.67	1	Pseudohyphae	+++	++	++	++	+	*Candida parapsilosis*
YHS182	HS61	M	2	0. 5	0. 125	4	+	+++	+++	+++	+++	-33.33	2	Pseudohyphae	+++	++	++	+	-	*Candida parapsilosis*
YHS183	HS61	M	2	0. 25	0. 125	0. 0625	-	+++	+++	++	++	-20	1	nd	++	++	++	++	+	*Candida parapsilosis*
YHS184	HS61	M	2	0. 25	0. 125	0. 031	-	+++	+++	+++	+++	-33.33	2	nd	+++	++	+	-	-	*Candida parapsilosis*
YHS185	HS61	M	2	0. 25	0. 125	0. 031	-	++	+++	+++	+++	-40	2	Pseudohyphae	+++	++	+	-	-	*Candida parapsilosis*
YHS186	HS61	M	2	0. 25	0. 125	0. 031	-	+++	++	++	++	-53.33	2	Pseudohyphae	+++	+++	+	+	-	*Candida parapsilosis*
YHS187	HS61	M	2	0. 125	0. 125	0. 0156	-	+	++	++	++	-20	1	nd	++	++	+	++	-	*Torulaspora delbrueckii*
YHS188	HS61	M	2	0. 125	0. 125	0. 0156	-	+++	+++	+++	+++	-40	1	nd	+++	++	-	-	-	*Candida parapsilosis*
YHS189	HS61	M	2	0. 5	0. 125	4	+	++	++	++	++	-40	0	nd	++	++	++	++	-	*Torulaspora delbrueckii*
YHS190	HS61	M	2	0. 25	0. 125	0. 031	-	+++	+++	+++	+++	-26.67	1	nd	+++	++	-	-	-	*Candida parapsilosis*
YHS191	HS61	M	2	0. 25	0. 125	0. 031	-	+++	+++	+++	+++	-33.33	2	Pseudohyphae	+++	++	++	++	+	*Candida parapsilosis*
YHS192	HS61	M	2	0. 25	0. 125	0. 031	-	+++	+++	+++	+++	-53.33	2	Pseudohyphae	+++	++	-	-	-	*Candida parapsilosis*
YHS193	HS61	M	2	0. 25	0. 125	0. 031	-	++	+++	+++	+++	-100	2	Pseudohyphae	+++	+	-	-	-	*Candida parapsilosis*
YHS194	HS62	F	4	0. 5	0. 125	0. 0625	-	-	++	++	++	-33.33	3	Hyphae	+++	+	+	+	-	*Trichosporon asahii*
YHS195	HS62	F	4	0. 5	0. 125	0. 0625	-	+++	++	++	++	-20	3	Hyphae	++	+	+	+	-	*Trichosporon asahii*
YHS196	HS62	F	4	2	0. 125	0. 031	-	+++	+++	+++	+++	-60	1	Pseudohyphae	+++	++	++	++	+	*Candida parapsilosis*
YHS197	HS62	F	4	0. 125	0. 125	0. 0156	-	+++	+++	+++	+++	-60	3	Pseudohyphae	+++	+	+	-	-	*Candida parapsilosis*
YHS198	HS62	F	4	0. 125	0. 125	0. 0156	-	+	+	+	-	-53.33	1	nd	+++	++	-	-	-	*Rhodotorula mucilaginosa*
YHS199	HS62	F	4	0. 5	0. 125	2	+	+++	+++	+++	+++	-36.67	0	nd	++	++	-	-	-	*Rhodotorula mucilaginosa*
YHS200	HS62	F	4	0. 125	0. 125	0. 0156	-	+++	+++	+++	+++	-60	3	Pseudohyphae	+++	++	+	+	+	*Candida parapsilosis*
YHS201	HS62	F	4	0. 5	0. 125	0. 0625	+	+++	+++	+++	+++	-60	3	Pseudohyphae	+++	+	+	+	-	*Candida parapsilosis*
YHS202	HS62	F	4	0. 125	0. 125	0. 0156	-	+	+++	++	++	-33.33	0	nd	++	-	-	-	-	*Rhodotorula mucilaginosa*
YHS203	HS62	F	4	0. 125	0. 125	0. 0156	-	+++	++	++	++	-40	3	Pseudohyphae	++	+	+	+	-	*Rhodotorula mucilaginosa*
YHS204	HS62	F	4	0. 5	0. 125	0. 0625	-	+++	++	++	++	-100	3	Pseudohyphae	+++	++	+	-	-	*Candida parapsilosis*
YHS205	HS62	F	4	0. 125	0. 125	0. 0156	-	+	-	-	-	20	0	nd	+	++	-	-	-	*Rhodotorula mucilaginosa*
YHS206	HS62	F	4	0. 5	0. 125	0. 031	+	+++	+	+	-	-26.67	0	nd	+	+++	-	-	-	*Rhodotorula mucilaginosa*
YHS207	HS62	F	4	1	0. 125	0. 0156	-	+++	+++	+++	+++	-60	3	Pseudohyphae	+++	+	+	+	-	*Candida parapsilosis*
YHS208	HS62	F	4	0. 125	0. 125	0. 0156	-	+	+	+	-	20	0	nd	+	+++	-	-	-	*Rhodotorula mucilaginosa*
YHS209	HS62	F	4	0. 125	0. 125	0. 0156	-	-	++	++	++	-6.67	1	nd	++	++	++	++	-	*Rhodotorula mucilaginosa*
YHS210	HS64	F	3	0. 125	0. 125	0. 0156	-	+++	++	++	++	-33.33	1	nd	+++	+++	-	-	-	*Pichia fermentans*
YHS211	HS64	F	3	0. 5	0. 125	0. 0156	+	+++	+++	+++	+++	-26.67	1	Hyphae	+++	+++	+++	+++	-	*Candida albicans*
YHS212	HS64	F	3	0. 5	0. 125	0. 0156	+	+++	+++	+++	+++	-26.67	1	nd	+++	+++	++	++	-	*Candida albicans*

YHS213	HS64	F	3	0. 5	0. 125	0. 25	+	+++	+++	+++	+++	-33.33	1	nd	+++	+++	+++	++	-	*Candida albicans*
YHS214	HS64	F	3	0. 5	0. 125	0. 031	+	+++	+++	+++	+++	-13.33	1	nd	+++	+++	+++	++	++	*Candida albicans*
YHS215	HS64	F	3	0. 5	0. 125	0. 25	+	+++	+++	+++	+++	-26.67	1	Hyphae	+++	+++	+++	++	-	*Candida albicans*
YHS216	HS64	F	3	0. 5	0. 125	0. 031	+	+++	+++	+++	+++	-13.33	1	nd	+++	+++	+++	++	-	*Candida albicans*
YHS217	HS65	M	5	0. 5	0. 125	0. 125	-	+++	+++	+++	+++	-46.67	1	nd	+++	+++	+++	++	-	*Candida lusitaniae*
YHS218	HS65	M	5	0. 5	0. 125	0. 125	+	+++	+++	+++	+++	-50	1	nd	+++	+++	+++	++	-	*Candida lusitaniae*
YHS219	HS65	M	5	0. 5	0. 125	0. 125	-	+	++	++	++	20	3	nd	++	-	-	-	-	*Candida pararugosa*
YHS220	HS65	M	5	0. 125	0. 125	0. 0156	-	++	+	+	+	20	0	nd	++	-	-	-	-	*Rhodotorula mucilaginosa*
YHS221	HS65	M	5	0. 25	0. 125	2	+	+	++	++	-	-13.33	0	nd	+++	+++	-	-	-	*Rhodotorula mucilaginosa*
YHS222	HS65	M	5	0. 125	0. 125	0. 0156	-	+	+++	+++	+++	-3.33	0	nd	+	-	-	-	-	*Rhodotorula mucilaginosa*
YHS223	HS65	M	5	0. 5	0. 125	0. 031	+	+++	+++	+++	+++	-13.33	0	nd	+++	+++	++	++	-	*Rhodotorula mucilaginosa*
YHS224	HS65	M	5	0. 125	0. 125	0. 0156	-	+++	+++	+++	+++	20	0	nd	+	+	-	-	-	*Rhodotorula mucilaginosa*
YHS225	HS65	M	5	0. 125	0. 125	0. 0156	-	+	++	++	++	-53.33	0	nd	++	++	++	++	-	*Rhodotorula mucilaginosa*
YHS226	HS65	M	5	0. 125	0. 125	0. 0156	-	+	+	+	-	20	0	nd	+	-	-	-	-	*Rhodotorula mucilaginosa*
YHS227	HS67	F	1	0. 125	0. 125	> 8	++	+++	-	-	-	-33.33	0	nd	+++	+++	-	-	-	*Torulaspora delbrueckii*
YHS228	HS67	F	1	0. 125	0. 125	0. 0156	-	+++	+++	-	-	6.67	0	nd	+	-	-	-	-	*Torulaspora delbrueckii*
YHS229	HS67	F	1	0. 125	0. 125	0. 0156	-	+	+	-	-	20	1	nd	+	-	-	-	-	*Rhodotorula mucilaginosa*
YHS230	HS67	F	1	> 64	0. 5	2	+	+++	-	-	-	-33.33	1	nd	+	++	-	-	-	*Rhodotorula mucilaginosa*
YHS231	HS67	F	1	0. 125	0. 125	0. 0156	-	+++	+++	+++	+++	-20	2	nd	++	++	++	++	-	*Torulaspora delbrueckii*
YHS232	HS67	F	1	0. 125	0. 125	0. 0156	-	+++	+++	+++	+++	-3.33	2	nd	++	++	++	++	-	*Torulaspora delbrueckii*
YHS233	HS67	F	1	0. 125	0. 125	0. 0156	+	+++	+++	+++	+++	-13.33	1	nd	+	+	-	-	-	*Torulaspora delbrueckii*
YHS234	HS67	F	1	0. 125	0. 125	0. 0156	-	-	-	-	-	-10	0	nd	-	-	-	-	-	*Torulaspora delbrueckii*
YHS235	HS67	F	1	0. 125	0. 125	0. 0156	-	-	-	-	-	0	0	nd	-	-	-	-	-	*Torulaspora delbrueckii*
YHS236	HS67	F	1	0. 125	0. 125	0. 0156	-	+++	+++	-	-	-13.33	0	nd	+++	+++	-	-	-	*Torulaspora delbrueckii*
YHS237	HS67	F	1	> 64	0. 125	2	+	+++	-	-	-	13.33	0	nd	+	+++	-	-	-	*Torulaspora delbrueckii*
YHS238	HS67	F	1	> 64	0. 125	0. 0156	-	+++	-	-	-	6.67	1	nd	+	+++	-	-	-	*Torulaspora delbrueckii*
YHS239	HS67	F	1	0. 125	0. 125	0. 0156	-	+++	+++	+++	+++	0	1	nd	+	++	-	-	-	*Torulaspora delbrueckii*
YHS240	HS67	F	1	0. 5	0. 125	4	+	+++	+++	+++	+++	-33.33	1	nd	+	++	-	-	-	*Rhodotorula mucilaginosa*
YHS241	HS67	F	1	0. 25	0. 125	0. 0156	-	+	+++	+++	+++	-20	1	nd	+	-	-	-	-	*Torulaspora delbrueckii*
YHS242	HS67	F	1	8	0. 125	2	+	+	-	-	-	0	0	nd	+++	+++	-	-	-	*Torulaspora delbrueckii*
YHS243	HS67	F	1	0. 125	0. 125	0. 0156	-	++	+	+	+	13.33	0	nd	+	+	-	-	-	*Torulaspora delbrueckii*
YHS244	HS68	F	4	0. 125	0. 125	0. 0156	-	++	+++	-	-	0	0	nd	+	-	-	-	-	*Torulaspora delbrueckii*
YHS245	HS68	F	4	0. 125	0. 125	0. 0156	-	++	++	++	++	-10	1	Hyphae	++	++	++	++	-	*Penicillium paneum*
YHS246	HS69	M	6	> 64	0. 125	2	+	+++	-	-	-	-26.67	1	Hyphae	+++	+++	+++	+++	-	*Aspergillus pseudoglaucus*
YHS247	HS70	F	11	> 64	0. 125	2	+	+++	-	-	-	-20	3	Hyphae	+++	+++	-	-	-	*Penicillium paneum*
YHS248	HS71	M	1	> 64	0. 125	2	+	+++	+++	+++	+++	-23.33	2	Hyphae	+++	+++	+++	++	-	*Candida albicans*

YHS249	HS73	F	4	> 64	0. 25	1	+	++	+++	+++	+++	-33.33	2	Hyphae	+++	+++	+++	++	-	*Candida albicans*
YHS250	HS74	M	6	> 64	0. 125	2	+	++	+++	+++	+++	-33.33	2	Hyphae	+++	+++	+++	++	-	*Candida albicans*
YHS251	HS75	F	1	> 64	0. 125	2	+	++	+++	+++	+++	-26.67	2	Hyphae	+++	+++	+++	++	+	*Candida albicans*
YHS252	HS76	M	1	> 64	0. 125	8	++	++	+++	+++	+++	-6.67	1	Hyphae	+++	+++	+++	++	-	*Aspergillus pseudoglaucus*
YHS253	HS77	F	4	0. 5	0. 25	1	+	+++	+++	+++	+++	-20	2	nd	+++	+++	++	++	-	*Candida albicans*
YHS254	HS79	M	0	64	0. 5	8	+	+++	+++	+++	+++	-33.33	2	Hyphae	+++	+++	+	+	+	*Candida albicans*
YHS255	HS80	F	0	> 64	0. 5	2	+	+++	+++	+++	+++	-20	2	Hyphae	+++	+++	+	++	++	*Candida albicans*
YHS256	HS81	F	7	0. 5	0. 5	1	+	+++	+++	+++	+++	-26.67	2	Hyphae	+++	+++	+++	++	-	*Candida albicans*
YHS257	HS84	F	6	> 64	0. 5	4	+	+++	+++	+++	+++	-20	2	Hyphae	+++	+++	++	++	-	*Candida albicans*
YHS258	HS85	F	10	0. 25	0. 125	> 8	++	+++	+++	+++	+++	-33.33	1	Hyphae	+++	+++	+++	++	-	*Candida albicans*
YHS259	HS85	F	10	0. 125	0. 125	8	++	+++	+++	+++	+++	-20	1	Hyphae	+++	+++	+++	++	-	*Candida albicans*
YHS260	HS85	F	10	0. 25	0. 125	> 8	++	++	+++	+++	+++	-20	2	Hyphae	+++	+++	+++	++	-	*Candida albicans*
YHS261	HS85	F	10	0. 25	0. 125	> 8	++	++	+++	+++	+++	-23.33	2	Hyphae	+++	+++	+++	++	-	*Candida albicans*
YHS262	HS85	F	10	0. 25	0. 125	> 8	+	++	+++	+++	+++	-23.33	1	Hyphae	+++	+++	+	+	+	*Candida albicans*
YHS263	HS85	F	10	4	0. 125	0. 25	+	+++	+++	+++	+++	-20	2	Hyphae	+++	+++	+	+	+	*Candida albicans*
YHS264	HS85	F	10	1	0. 125	1	++	+++	+++	+++	+++	-23.33	1	Hyphae	++	+++	+++	+++	-	*Candida albicans*
YHS265	HS85	F	10	0. 125	0. 125	2	++	+++	+++	+++	+++	-26.67	1	Hyphae	+++	+++	++	++	-	*Candida albicans*
YHS266	HS85	F	10	0. 125	0. 125	> 8	++	+++	+++	+++	+++	-6.67	2	Hyphae	+++	+++	++	++	-	*Candida albicans*
YHS267	HS85	F	10	0. 125	0. 125	8	++	+++	+++	+++	+++	-13.33	2	Hyphae	+++	+++	++	++	-	*Candida albicans*
YHS268	HS85	F	10	0. 125	0. 125	> 8	++	+++	+++	+++	+++	-13.33	2	Hyphae	+++	+++	++	++	-	*Candida albicans*
YHS269	HS85	F	10	> 64	0. 125	> 8	++	+++	+++	+++	+++	-20	2	Hyphae	+++	+++	+++	+++	-	*Candida albicans*
YHS270	HS85	F	10	0. 25	0. 125	> 8	+	+++	+++	+++	+++	-26.67	1	nd	+++	+++	++	+	-	*Candida albicans*
YHS271	HS85	F	10	0. 125	0. 125	> 8	++	+++	+++	+++	+++	-26.67	1	nd	+++	+++	++	+++	-	*Candida albicans*
YHS272	HS85	F	10	4	0. 125	0. 0625	++	+++	+++	+++	+++	-26.67	2	Hyphae	+++	+++	+++	++	-	*Candida fermentati*
YHS273	HS85	F	10	0. 125	0. 125	> 8	++	+++	+++	+++	+++	-26.67	2	Hyphae	+++	+++	+++	++	-	*Candida albicans*
YHS274	HS85	F	10	0. 125	0. 125	8	++	+++	+++	+++	+++	-26.67	2	Hyphae	+++	+++	+++	++	-	*Candida albicans*
YHS275	HS85	F	10	0. 125	0. 125	> 8	++	+++	+++	+++	+++	-20	2	Hyphae	+++	+++	+++	++	-	*Candida albicans*
YHS276	HS85	F	10	0. 25	0. 125	2	++	+++	+++	+++	+++	-46.67	2	Hyphae	+++	+++	+++	++	-	*Candida albicans*
YHS277	HS85	F	10	0. 5	0. 125	0. 0625	+	+++	+++	+++	+++	-33.33	2	Hyphae	+++	+++	+++	+	-	*Candida albicans*
YHS278	HS85	F	10	0. 25	0. 125	> 8	++	+++	+++	+++	+++	-20	2	Hyphae	+++	+++	+++	+++	-	*Candida albicans*
YHS279	HS85	F	10	0. 125	0. 125	0. 25	+	+++	+++	+++	+++	-20	2	Hyphae	+++	+++	+++	++	-	*Candida albicans*
YHS280	HS85	F	10	0. 125	0. 125	0. 125	++	+++	+++	+++	+++	-23.33	2	Hyphae	+++	+++	+++	++	-	*Candida albicans*
YHS281	HS85	F	10	0. 25	0. 125	2	++	+++	+++	+++	+++	-26.67	2	Hyphae	+++	+++	+++	++	-	*Candida albicans*
YHS282	HS85	F	10	0. 5	0. 125	2	++	+++	+++	+++	+++	-6.67	2	Hyphae	+++	+++	+++	++	-	*Candida albicans*
YHS283	HS86	M	7	0. 5	0. 5	0. 125	++	+++	+++	+++	+++	-33.33	2	Hyphae	+++	+++	+++	+++	-	*Candida albicans*
YHS284	HS87	M	9	0. 125	0. 125	0. 031	-	-	+++	+++	+++	20	1	nd	+	+	+	-	-	*Cryptococcus saitoi*

YHS285	HS87	M	9	> 64	0. 125	0. 0156	-	-	+++	+++	+++	-13.33	2	Hyphae	++	++	++	+	-	*Eurotium rubrum*
YHS286	HS88	M	7	> 64	0. 125	2	++	+++	+++	+++	+++	-26.67	2	Hyphae	+++	+++	+++	++	-	*Candida albicans*
YHS287	HS89	M	12	0. 5	0. 125	0. 125	++	+++	+++	+++	+++	-46.67	2	Hyphae	+++	+++	+++	++	-	*Candida albicans*
YHS288	HS89	M	12	0. 5	0. 125	2	+	+++	+++	+++	+++	-33.33	1	nd	+++	+++	+++	++	-	*Candida albicans*
YHS289	HS90	F	8	0. 25	0. 5	2	++	+++	+++	+++	+++	-26.67	2	Hyphae	+++	+++	+++	++	-	*Candida albicans*
YHS290	HS91	F	2	0. 25	0. 5	0. 25	++	+++	+++	+++	+++	-40	2	Pseudohyphae	+++	+++	+++	+	-	*Candida parapsilosis*
YHS291	HS91	F	2	0. 5	0. 125	0. 25	-	+++	+++	+++	+++	-46.67	2	Pseudohyphae	+++	+++	+++	++	-	*Candida parapsilosis*
YHS292	HS91	F	2	> 64	0. 125	> 8	++	++	+++	+++	+++	13.33	2	Hyphae	+++	++	+	-	-	*Eurotium amstelodami*
YHS293	HS91	F	2	0. 25	0. 125	> 8	-	+++	++	++	++	-46.67	2	Pseudohyphae	+++	++	+	-	-	*Candida parapsilosis*
YHS294	HS92	F	12	0. 5	0. 125	> 8	+	++	+++	+++	+++	-6.67	3	Pseudohyphae	+++	+++	+++	+	-	*Saccharomyces cerevisiae*
YHS295	HS92	F	12	0. 25	1	4	++	+++	+++	+++	+++	-20	2	Hyphae	+++	+++	++	++	-	*Penicillium brevicompactum*
YHS296	HS93	F	4	> 64	0. 5	2	+	+++	+++	+++	+++	-33.33	3	Hyphae	+++	+++	+++	++	-	*Aspergillus cristatus*
YHS297	HS94	F	4	0. 5	0. 125	1	+	+++	+++	+++	+++	-33.33	1	nd	+++	++	+++	++	-	*Candida albicans*
YHS298	HS95	F	10	0. 125	0. 125	4	++	+++	+++	+++	+++	-26.67	2	Hyphae	+++	+++	+++	++	-	*Candida albicans*
YHS299	HS96	F	12	0. 5	0. 125	1	+	+++	+++	+++	+++	-26.67	2	Hyphae	+++	+++	+++	++	-	*Candida albicans*
YHS300	HS97	M	6	> 64	0. 5	2	++	+++	+++	+++	+++	-26.67	2	Hyphae	+++	+++	+++	++	-	*Candida albicans*
YHS301	HS98	F	16	> 64	1	> 8	+	+++	+++	+++	+++	-20	0	nd	+++	++	+++	++	-	*Candida parapsilosis*
YHS302	HS99	F	3	0. 5	0. 125	> 8	++	++	+++	+++	+++	-53.33	2	Pseudohyphae	+++	++	+	-	-	*Candida parapsilosis*
YHS303	HS99	F	3	0. 5	0. 125	0. 031	++	+++	+++	+++	+++	-53.33	2	Pseudohyphae	+++	+++	+	+	-	*Candida parapsilosis*
YHS304	HS100	M	0	0. 5	0. 5	2	++	+++	+++	+++	+++	4.94	2	Hyphae	+++	+++	+++	++	-	*Candida albicans*
YHS305	HS101	M	4	0. 125	0. 125	0. 0156	-	+++	+++	+++	+++	23.46	3	Hyphae	+++	+++	-	-	-	*Candida albicans*
YHS306	HS102	F	13	0. 25	0. 125	> 8	++	++	-	-	-	-60	0	nd	++	+	-	-	-	*Rhodotorula mucilaginosa*
YHS307	HS102	F	13	0. 125	0. 125	4	++	+++	+	+	+	-56.67	3	Hyphae	+++	+++	+	-	-	*Candida pararugosa*
YHS308	HS102	F	13	0. 5	0. 125	0. 0625	+	++	+	+	+	-100	3	Hyphae	+++	++	+	-	-	*Mucor circinelloides*
YHS309	HS102	F	13	4	0. 125	0. 0156	+	+	+++	+++	+++	20	3	Hyphae	++	+	+	-	-	*Pleurostomophora richardsiae*
YHS310	HS103	M	7	> 64	0. 125	0. 031	+	++	++	++	++	-100	3	Hyphae	+++	++	+++	++	-	*Mucor circinelloides*
YHS311	HS104	M	4	> 64	1	4	++	+++	+++	+++	+++	17.28	2	Hyphae	+++	+++	+++	++	-	*Candida albicans*
YHS312	HS105	F	8	> 64	0. 125	> 8	+	+++	+++	+++	+++	-33.33	1	Pseudohyphae	+++	+++	+++	++	-	*Candida parapsilosis*
YHS313	HS105	F	8	> 64	0. 125	4	++	+++	+++	+++	+++	-33.33	2	Hyphae	+++	+++	+++	++	-	*Candida albicans*
YHS314	HS105	F	8	> 64	0. 125	2	+	+++	+++	+++	+++	-33.33	1	Hyphae	+++	+++	+++	+++	-	*Candida albicans*
YHS315	HS105	F	8	> 64	0. 125	4	+	+++	+++	+++	+++	-33.33	0	nd	+++	+++	+++	++	-	*Candida albicans*
YHS316	HS105	F	8	> 64	0. 125	4	+	+++	+++	+++	+++	-26.67	0	nd	+++	+++	+++	++	-	*Candida albicans*
YHS317	HS105	F	8	> 64	0. 125	0. 25	+	+++	+++	+++	+++	-100	0	nd	+++	+++	+++	+	-	*Candida metapsilosis*
YHS318	HS105	F	8	> 64	0. 125	2	++	+++	+++	+++	+++	-20	0	nd	+++	+++	+++	++	-	*Candida albicans*
YHS319	HS105	F	8	> 64	0. 125	> 8	+	+++	+++	+++	++	-20	2	Hyphae	+++	+++	+++	++	-	*Candida albicans*
YHS320	HS105	F	8	> 64	0. 125	8	++	+++	+++	+++	+++	-33.33	1	Hyphae	+++	+++	+++	++	-	*Candida albicans*

YHS321	HS105	F	8	32	0. 25	> 8	++	+++	+++	+++	++	-26.67	2	Hyphae	+++	+++	+++	++	+	*Candida albicans*
YHS322	HS105	F	8	> 64	0. 125	2	+	+++	+++	+++	+++	-20	2	Hyphae	+++	+++	+++	+++	-	*Candida albicans*
YHS323	HS105	F	8	> 64	0. 125	2	+	+++	+++	+++	+++	-33.33	1	Hyphae	+++	+++	+++	++	-	*Candida albicans*
YHS324	HS105	F	8	> 64	0. 125	2	+	+++	+++	+++	+++	-26.67	1	Hyphae	+++	+++	+++	+++	-	*Candida albicans*
YHS325	HS105	F	8	> 64	0. 125	4	+	+++	+++	+++	+++	-33.33	1	Hyphae	+++	+++	+++	+++	-	*Candida albicans*
YHS326	HS105	F	8	> 64	0. 125	8	+	+++	+++	+++	+++	-33.33	1	Hyphae	+++	+++	+++	++	-	*Candida albicans*
YHS327	HS105	F	8	> 64	1	8	+	+++	+++	+++	+++	-33.33	1	Hyphae	+++	+++	+++	++	-	*Candida albicans*
YHS328	HS105	F	8	> 64	0. 125	8	++	+++	+++	+++	+++	-26.67	1	Hyphae	+++	+++	+++	++	-	*Candida albicans*
YHS329	HS105	F	8	64	0. 125	8	++	+++	+++	+++	+++	-26.67	2	Hyphae	+++	+++	++	++	-	*Candida albicans*
YHS330	HS105	F	8	32	0. 125	4	+	+++	+++	+++	+++	-26.67	2	Hyphae	+++	+++	+++	+++	-	*Candida albicans*
YHS331	HS105	F	8	> 64	0. 125	0. 0156	++	+++	+++	+++	+++	-33.33	2	Hyphae	+++	+++	+++	+++	-	*Candida albicans*
YHS332	HS105	F	8	> 64	0. 125	0. 0156	++	+++	+++	+++	+++	-33.33	2	Hyphae	+++	+++	+++	+++	-	*Candida albicans*
YHS333	HS106	F	5	> 64	0. 125	8	++	+++	++	++	++	-33.33	0	nd	+	+++	+	-	-	*Rhodotorula mucilaginosa*
YHS334	HS106	F	5	0. 125	0. 125	0. 031	-	+	+	+	+	-100	0	nd	+	++	+	-	-	*Rhodotorula mucilaginosa*
YHS335	HS106	F	5	0. 25	0. 125	0. 031	++	+	+	+	+	-100	0	nd	+	+	+	-	-	*Rhodotorula mucilaginosa*
YHS336	HS106	F	5	0. 125	0. 125	0. 0156	-	+	+	+	+	-100	0	nd	+	+	+	-	-	*Rhodotorula mucilaginosa*
YHS337	HS106	F	5	> 64	0. 125	4	+	++	-	-	-	-100	0	nd	+	+	-	-	-	*Rhodotorula mucilaginosa*
YHS338	HS106	F	5	> 64	0. 125	0. 0156	-	++	+	+	+	-100	0	nd	+++	+++	+	+	-	*Rhodotorula mucilaginosa*
YHS339	HS106	F	5	> 64	0. 125	0. 0156	++	+++	++	++	++	-100	0	nd	+	+	+	-	-	*Rhodotorula mucilaginosa*
YHS340	HS106	F	5	> 64	0. 125	0. 0156	+	+	++	++	++	-100	0	nd	+	+	+	-	-	*Rhodotorula mucilaginosa*
YHS341	HS106	F	5	> 64	0. 125	0. 0156	++	+	++	++	++	-100	0	nd	++	+	+	+	-	*Rhodotorula mucilaginosa*
YHS342	HS106	F	5	> 64	0. 5	0. 25	-	+	++	++	++	-100	0	nd	+	+	+	-	-	*Rhodotorula mucilaginosa*
YHS343	HS106	F	5	> 64	0. 125	0. 0156	++	+	+	+	+	-100	0	nd	++	+	+	-	-	*Rhodotorula mucilaginosa*
YHS344	HS106	F	5	> 64	0. 125	0. 0156	-	+	+	+	+	-100	0	nd	+++	+++	-	-	-	*Rhodotorula mucilaginosa*
YHS345	HS106	F	5	> 64	0. 125	0. 0156	-	++	++	++	++	-100	0	nd	+	-	-	-	-	*Rhodotorula mucilaginosa*
YHS346	HS106	F	5	> 64	0. 125	0. 0156	-	+++	++	++	++	-100	0	nd	+++	+	+	-	-	*Rhodotorula mucilaginosa*
YHS347	HS106	F	5	> 64	0. 125	2	++	++	++	++	++	-100	0	nd	++	+	-	-	-	*Rhodotorula mucilaginosa*
YHS348	HS106	F	5	> 64	0. 125	0. 0156	-	+	++	++	++	-100	0	nd	++	+	+	-	-	*Rhodotorula mucilaginosa*
YHS349	HS106	F	5	> 64	0. 125	0. 0156	-	++	++	++	++	-100	0	nd	++	++	+	-	-	*Rhodotorula mucilaginosa*

*calculated as the deviation of the inhibition halo diameter (Ø) from that of the M28-4D *S. cerevisiae* strain, according to the following formula: (Ø sample – Ø M284D strain) / Ø M284D strain *100. #, 0= non-invasive; 1= poor invasive; 2= invasive; 3= very invasive. -, no growth as measured by $OD_{630} \leq 0.2$ or cfu/ml $\leq 10^5$; +, poor growth as measured by $0.2 < OD_{630} \leq 0.7$ or $10^5<$ cfu/ml$\leq 10^6$; ++ good growth as measured by $0.7 < OD_{630} \leq 1.2$ or $10^6<$ cfu/ml$\leq 10^7$; +++, very good growth as measured by $OD_{630} > 1.2$ or cfu/ml$> 10^7$. na, not applicable; nd, not detected
