# Supplementary material for: Age and Gender Affect the Composition of Fungal Population of the Human Gastrointestinal Tract
Source: Front Microbiol. 2016 Aug 3;7:1227. doi: 10.3389/fmicb.2016.01227 (PMC4971113; doi:10.3389/fmicb.2016.01227)
Supplement: Supplementary Table S3 — Permutational multivariate analysis of variance (PERMANOVA) tests on unweighted and weighted UniFrac distances and Bray-Curtis dissimilarity. [file Table3.PDF]

Table S3: Permutational multivariate analysis of variance (PERMANOVA) tests on unweighted and weighted UniFrac distances and Bray-Curtis dissimilarity.

	**Metric**	**F**	$\mathbf{R^2}$	***p*-value**
between genders	unweighted Unifrac	3.15	0.054	0.033
	weighted Unifrac	2.48	0.043	0.07
	Bray-Curtis	4.80	0.08	0.001
among age groups	unweighted Unifrac	1.79	0.092	0.031
	weighted Unifrac	1.27	0.067	0.268
	Bray-Curtis	1.69	0.087	0.11
