# Supplementary material for: Age and Gender Affect the Composition of Fungal Population of the Human Gastrointestinal Tract
Source: Front Microbiol. 2016 Aug 3;7:1227. doi: 10.3389/fmicb.2016.01227 (PMC4971113; doi:10.3389/fmicb.2016.01227)
Supplement: Supplementary Table S4 — Mean relative abundance (%) of OTUs at the genus level of fungal gut microbiota of healthy subjects from metagenomics analysis. [file Table4.PDF]

Table S4: Mean relative abundance (%) of OTUs at the genus level of fungal gut microbiota of healthy subjects from metagenomics analysis.

	mean relative abundance (%)						
**Taxonomy**	**Total**	**Male**	**Female**	**Infants**	**Children**	**Adolescents**	**Adults**
Penicillium	22.362	25.184	19.637	22.041	20.558	12.233	29.652
Aspergillus	22.202	37.509	7.423	35.442	33.227	11.552	5.156
Candida	16.918	10.975	22.657	11.963	12.165	20.226	24.427
Fungi_unidentified_1_1	6.330	6.716	5.958	2.731	8.525	3.110	7.123
Blastocystis	5.311	0.000	10.439	0.000	0.000	13.630	11.393
Pichia	3.485	0.045	6.806	0.126	0.004	11.382	6.248
Mucor	3.057	0.885	5.154	0.233	3.317	12.196	0.080
Debaryomyces	2.979	1.322	4.578	3.644	1.683	2.065	4.693
Malassezia	2.871	2.541	3.190	1.458	3.145	3.608	3.001
Ascomycota_unidentified_1_1	2.133	3.148	1.153	6.803	0.813	0.862	1.692
Eremothecium	1.671	0.010	3.274	0.039	3.671	0.000	0.829
Eurotiomycetes_unidentified_1	1.500	2.690	0.352	7.065	0.459	0.534	0.029
Tremellomycetes_unidentified_1	1.200	2.190	0.245	2.167	1.816	0.680	0.080
Cyberlindnera	0.841	0.108	1.548	0.155	0.724	0.024	1.778
Mucoraceae_unidentified	0.795	0.007	1.555	0.039	1.970	0.024	0.080
Saccharomyces	0.713	1.243	0.201	0.301	0.437	3.122	0.177
Ustilago	0.622	1.062	0.198	0.428	1.391	0.073	0.000
Nectriaceae_unidentified	0.469	0.580	0.362	1.623	0.230	0.000	0.320
Rhodotorula	0.450	0.132	0.757	0.437	0.517	0.595	0.303
Malasseziales_unidentified_1	0.385	0.222	0.543	0.010	0.464	0.012	0.680
Wallemia	0.298	0.132	0.459	0.097	0.552	0.279	0.097
Xeromyces	0.290	0.569	0.020	0.010	0.747	0.000	0.000
Trichosporon	0.247	0.496	0.007	1.390	0.009	0.000	0.000
Preussia	0.237	0.482	0.000	0.000	0.614	0.000	0.000
Pleosporales_unidentified_1	0.188	0.236	0.141	0.068	0.084	0.790	0.109
Phoma	0.174	0.035	0.308	0.097	0.278	0.012	0.160
Trichocomaceae_unidentified	0.174	0.021	0.322	0.049	0.031	0.000	0.514
Aureobasidium	0.167	0.236	0.101	0.000	0.221	0.583	0.000
Botrytis	0.162	0.014	0.305	0.194	0.186	0.061	0.160
Ustilaginomycetes_unidentified_1	0.095	0.010	0.178	0.000	0.000	0.680	0.000
Helotiales_unidentified_1	0.085	0.174	0.000	0.000	0.000	0.607	0.000
Saccharomycetales_unidentified_1	0.085	0.010	0.158	0.117	0.119	0.000	0.063
Talaromyces	0.080	0.017	0.141	0.311	0.022	0.000	0.057
Ustilaginaceae_unidentified	0.080	0.007	0.151	0.000	0.208	0.000	0.000
Rhizopus	0.080	0.024	0.134	0.000	0.053	0.000	0.200
Cryptococcus	0.078	0.017	0.137	0.049	0.000	0.000	0.234
Torulaspora	0.077	0.087	0.067	0.039	0.066	0.255	0.029
Cordyceps	0.075	0.021	0.127	0.000	0.159	0.097	0.000
Sarcosomataceae_unidentified	0.072	0.000	0.141	0.000	0.186	0.000	0.000
Urocystis	0.072	0.146	0.000	0.000	0.186	0.000	0.000
Incertae_sedis_12_unidentified	0.070	0.000	0.137	0.000	0.141	0.000	0.051
Davidiella	0.056	0.021	0.090	0.049	0.062	0.012	0.074
Helminthosporium	0.053	0.000	0.104	0.000	0.137	0.000	0.000

Exophiala	0.051	0.073	0.030	0.204	0.035	0.012	0.000
Wickerhamomyces	0.044	0.017	0.070	0.049	0.000	0.000	0.120
Phaeosphaeriaceae_unidentified	0.039	0.000	0.077	0.000	0.000	0.000	0.131
Basidiomycota_unidentified_1_1	0.038	0.045	0.030	0.068	0.053	0.036	0.000
Tilletia	0.038	0.062	0.013	0.155	0.018	0.024	0.000
Ascosphaera	0.036	0.073	0.000	0.000	0.088	0.000	0.006
Ustilaginaceae_unidentified	0.034	0.000	0.067	0.000	0.088	0.000	0.000
Hypocreales_unidentified_1	0.031	0.042	0.020	0.029	0.009	0.146	0.006
Rasamsonia	0.029	0.031	0.027	0.039	0.027	0.000	0.040
Periconia	0.027	0.056	0.000	0.000	0.071	0.000	0.000
Incertae_sedis_25_unidentified	0.027	0.007	0.047	0.000	0.049	0.024	0.017
Udeniomyces	0.027	0.000	0.054	0.000	0.071	0.000	0.000
Dioszegia	0.026	0.007	0.044	0.019	0.053	0.012	0.000
Thermomyces	0.026	0.031	0.020	0.087	0.000	0.000	0.034
Sporobolomyces	0.020	0.035	0.007	0.058	0.022	0.012	0.000
Lophiostoma	0.017	0.035	0.000	0.000	0.000	0.121	0.000
Embellisia	0.015	0.028	0.003	0.000	0.004	0.097	0.000
Saccharomycetaceae_unidentified	0.015	0.003	0.027	0.010	0.000	0.000	0.046
Guehomyces	0.015	0.007	0.023	0.019	0.031	0.000	0.000
Puccinia	0.012	0.010	0.013	0.010	0.018	0.024	0.000
Pseudozyma	0.012	0.003	0.020	0.010	0.000	0.000	0.034
Ustilaginales_unidentified_1	0.012	0.024	0.000	0.000	0.009	0.061	0.000
Diatrypaceae_unidentified	0.009	0.017	0.000	0.000	0.022	0.000	0.000
Leucosporidiella	0.009	0.017	0.000	0.049	0.000	0.000	0.000
Endoconidioma	0.007	0.000	0.013	0.000	0.018	0.000	0.000
Chaetothyriales_unidentified_1	0.007	0.007	0.007	0.019	0.000	0.024	0.000
Tetracladium	0.007	0.014	0.000	0.000	0.000	0.049	0.000
Kluyveromyces	0.007	0.000	0.013	0.000	0.000	0.000	0.023
Diplodia	0.005	0.000	0.010	0.000	0.013	0.000	0.000
Sarcinomyces	0.005	0.000	0.010	0.000	0.013	0.000	0.000
Gymnoascus	0.005	0.000	0.010	0.000	0.013	0.000	0.000
Kazachstania	0.005	0.000	0.010	0.000	0.000	0.000	0.017
Arthrinium	0.005	0.000	0.010	0.000	0.013	0.000	0.000
Erythrobasidium	0.005	0.000	0.010	0.000	0.013	0.000	0.000
Dothideomycetes_unidentified_1	0.003	0.003	0.003	0.000	0.000	0.012	0.006
Mycocentrospora	0.003	0.000	0.007	0.000	0.009	0.000	0.000
Knufia	0.003	0.007	0.000	0.000	0.009	0.000	0.000
Hanseniaspora	0.003	0.000	0.007	0.000	0.000	0.000	0.011
Myrothecium	0.003	0.000	0.007	0.000	0.009	0.000	0.000
Sordariomycetes_unidentified_1	0.003	0.000	0.007	0.000	0.009	0.000	0.000
Taphrina	0.003	0.003	0.003	0.000	0.009	0.000	0.000
Wojnowicia	0.002	0.003	0.000	0.000	0.004	0.000	0.000
Podosphaera	0.002	0.000	0.003	0.000	0.004	0.000	0.000
Schizosaccharomyces	0.002	0.003	0.000	0.000	0.000	0.012	0.000
Coniochaeta	0.002	0.000	0.003	0.000	0.000	0.000	0.006
Claviceps	0.002	0.000	0.003	0.000	0.000	0.012	0.000
Sordariaceae_unidentified	0.002	0.003	0.000	0.000	0.004	0.000	0.000

Sakaguchia	0.002	0.003	0.000	0.000	0.000	0.012	0.000
Exobasidiales_unidentified_1	0.002	0.003	0.000	0.000	0.004	0.000	0.000
Quambalaria	0.002	0.000	0.003	0.000	0.000	0.000	0.006
Filobasidium	0.002	0.000	0.003	0.000	0.000	0.000	0.006
Hannaella	0.002	0.000	0.003	0.000	0.004	0.000	0.000
Lichtheimia	0.002	0.000	0.003	0.000	0.004	0.000	0.000
